# Toward a scientific understanding of the effectiveness, material basis and prescription compatibility of a Chinese herbal formula Dan-hong injection

**DOI:** 10.1038/srep46266

**Published:** 2017-04-10

**Authors:** Panlin Li, Weiwei Su, Sha Yun, Yiqiu Liao, Yinyin Liao, Hong Liu, Peibo Li, Yonggang Wang, Wei Peng, Hongliang Yao

**Affiliations:** 1 Guangdong Engineering and Technology Research Center for Quality and Efficacy Re-evaluation of Post-marketed TCM, Guangdong Key Laboratory of Plant Resources, School of Life Sciences, Sun Yat-sen University, Guangzhou 510275, P R. China

## Abstract

Since traditional Chinese medicine (TCM) is a complex mixture of multiple components, the application of methodologies for evaluating single-components Western medicine in TCM studies may have certain limitations. Appropriate strategies that recognize the integrality of TCM and connect to TCM theories remain to be developed. Here we use multiple unique approaches to study the scientific connotation of a TCM formula Dan-hong injection (DHI) without undermining its prescription integrity. The blood circulation improving and healing promoting effects of DHI were assessed by a qi stagnation blood stasis rat model and a mouse model of laser irradiation induced cerebral microvascular thrombosis. By UFLC-PDA-Triple Q-TOF-MS/MS and relevance analysis between chemical characters and biological effects, 82 chemical constituents and nine core components, whose blood circulation promoting effects were found comparable to that of whole DHI, were successfully identified. What’s more, the rationality of DHI prescription compatibility could be reflected not only in the maximum efficacy of the original ratio, but also in the interactions of compounds from different ingredient herbs, such as complementary activities and facilitating tissues distribution. This study provides scientific evidences in explanation of the clinical benefits of DHI, and also gives a good demonstration for the comprehensive evaluation of other TCM.

It has been well claimed that traditional Chinese medicine (TCM) elicits effects via a multi-compounds mechanism and fits well for the requirement in the therapy of complex diseases^1^,^2^,^3^,^4^. For a long period, the studies of TCM use the experience of western medicine researches for reference and achieved certain successes on discovery of new drugs^5^. However, the integrity of TCM and connection with TCM theories have been somewhat overlooked, which impose constraints on the explanation of some key problems, such as how to define TCM’s pharmacodynamic characteristics, how to identify the chemical material basis and pharmacologically active components, and how to clarify the rationality of TCM prescription composition rules.

Pharmacodynamic characteristics provide the basis for the rational therapeutic use of TCM and its further scientific researches. Different from the viewpoints about etiology and pathology in western medicine that one or more crucial pathogenetic factors lead to the development of a disease, TCM regards a disease as the combined influence of pathogenetic factors and maladjustments in body^6^. Namely, TCM treatment concentrates on the specific overall functional state of a patient, also referred to as pattern or syndrome (‘Zheng’ in Chinese). Therefore, the pharmacodynamic characteristics of TCM should be evaluated by model that reflects multi-system changes and parameters represent the general functional state.

Behind the specific pharmacodynamic characteristic of TCM are the bioactivities of many chemical compounds. However, most compounds in TCM are still unknown and not clear whether have biological activities or not. Previous efforts to discover the chemical constituents and bioactive components are mainly through isolating single compound from TCM and screening for bioactivity. However, the therapeutic effects of TCM are produced by complex interactions among its hundreds of chemical compounds^7^. Bioactive tests of single isolated compound may lead to the neglect of certain connections with other ingredients in the actual situation *in vivo*, and usually cannot achieve the expected activity^1^,^5^. In recent years, an increasing number of scientists have come to recognize that most herbal medicines may exert their therapeutic effects through the synergy of diverse components rather than a single compound. Therefore, the bioactive components should be explored by regarding the herb or TCM formula as an organic whole in which different compounds interact.

Prescription is the main form of TCM’s clinical use. The combined pharmacodynamic, pharmacokinetic, and toxicity-modulating effects of multiple components from ingredient herbs are considered to be crucial for the therapeutic effect of a TCM. It is worth noting that the contents of some compounds in TCM are very low, even below their effective concentration against specific pathogenetic factors, let alone the levels *in vivo* after administration. Therefore, there must be synergistic and/or additive effects between different compounds and different ingredient herbs. Additionally, the toxicity and side effect of single compound may be counteracted when used in formulation^8^. Revealing the scientificity and rationality of TCM prescription composition rules is an important topic in the progress of uncovering the mystery of TCM and increasing its application.

A number of TCM prescriptions although have achieved good clinical outcomes, the lack of scientific evidence would give rise to the underestimation of their value in modern medicine. Dan-hong injection (DHI) is a well-established TCM prescription developed based on years of clinical experiences. It consists of two commonly used herbal medicines *Salvia miltiorrhiza* (SM, Danshen in Chinese) and *Carthamus tinctorious* (CT, Honghua in Chinese). DHI has been applied extensively in clinical practice and shown significant therapeutic effects in patients with coronary heart disease^9^,^10^,^11^, cerebral thrombosis^12^,^13^,^14^. and hepatic veno-occlusive disease^15^. And it is most praiseworthy that DHI has a good safety record which the incidence rate of adverse drug reactions was 3.50‰^16^. Previous studies showed that its pharmacological mechanisms might involve promoting blood circulation, resolving stasis, vasodilatory, lipid-lowering anti-inflammation and antioxidation^12^,^17^,^18^,^19^. However, due to the indeterminate relationship between pharmacological activities, chemical constituents and ingredient herbs, the clinical application and quality control of DHI still faced big challenges.

In the present study, we carried out further study on DHI by strategies that recognize its formula integrity and connect to TCM theories. Firstly, the pharmacological characteristics of DHI were comprehensively assessed by a rat model with qi stagnation blood stasis syndrome and a mouse model of cerebral microvascular thrombosis induced by laser irradiation. Then in order to explore the material basis of its pharmacological effects, the chemical profile was systematically analyzed by ultra-fast liquid chromatography coupled with photo-diode array detector and quadrupole/time-of-flight mass spectrometry (UFLC-PDA-Triple Q-TOF-MS/MS) as a prerequisite. The core bioactive components contributing to the blood circulation promoting effect of DHI was identified based on Relevance Analysis between Chemical Characters and Biological Effects (RACCBE) approach which was proposed by our laboratory recently and has been successfully applied to uncover the bioactive chemical basis contribute to a certain pharmacological effect in Kou-yan-qing granule^20^, Compound xue-shuan-tong capsule^21^ and Shen-qi-fu-zheng injection^22^. The RACCBE approach used in this study mainly includes the following four steps: (1) nine prescription modified DHI samples were prepared with different proportions of SM and CT according to a two-factor, nine-level uniform design; (2) the nine DHI modified samples were tested by UPLC fingerprints and 13 main components were selected as chemical characters for relevance analysis; (3) aggregative parameters reflect hemorheological properties and blood coagulation function of the nine DHI modified samples were obtained by using the qi stagnation blood stasis rat model; (4) a combination of different mathematical methods, including grey relational analysis, multiple linear regression analysis and radial basis function analysis were performed to dissect the relevance between chemical constituents and bioactivities. The core bioactive components were determined by evaluating the contribution of each component, which is followed by an experimental validation of activity. And finally, the relationship between the two ingredient herbs SW and CT, and the rationality of prescription compatibility was further discussed.

## Results

### Effect of DHI on hemorheological properties and blood coagulation function in qi stagnation and blood stasis rats

**Figure 1:**
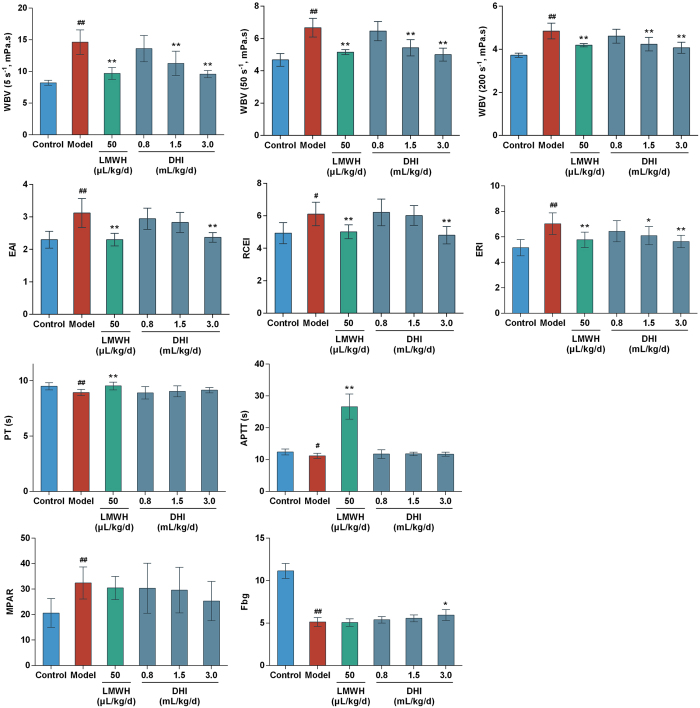
Effects of DHI on hemorheological properties and blood coagulation function in Qi stagnation and blood stasis rats. The influences of DHI on hemorheological properties were evaluated by whole blood viscosity (WBV) at different shear rates (5, 50 and 200 s^–1^), erythrocyte aggregation index (EAI), red corpuscle electrophoresis index (RCEI) and erythrocyte rigidity index (ERI). The effects of DHI on blood coagulation function were assessed by activated partial thromboplastin time (APTT), prothrombin time (PT), maximum platelet aggregation rate (MPAR) and fibrinogen levels (Fbg). LMWH: low molecular weight heparin; DHI: Dan-hong injection. Data are presented as mean ± standard deviation (n = 10). Pound signs indicate data that differed significantly from control (^#^P < 0.05, ^##^P < 0.01). Asterisks indicate data that differed significantly from model (*P < 0.05, **P < 0.01).

The rat model used in this study has been published previously by our team^21^. The qi stagnation and blood stasis rat model was generated by placing rats into ice water during the interval between adrenaline (Adr) injections, which led to the disturbance of whole body blood circulation. Based on the TCM theory that cold stimulation could block qi movement, ice water exposure was used to mimic the qi stagnation state in rat. Qi stagnation could cause blood stasis, and the maladjustment was aggravated when coupled with Adr’s effects on increasing heart rate, force of heart contraction and skin vasoconstriction. The parameters represent systematic blood circulation and blood coagulation were detected. More specifically, whole blood viscosity (WBV) reflects the intrinsic resistance of blood flow in the vasculature^23^. Erythrocyte aggregation index (EAI) and red corpuscle electrophoresis index (RCEI) both reflect the degree of aggregation among red blood cell (RBC). The increasing erythrocyte rigidity index (ERI) is associated with the reduced red blood cell deformability. Activated partial thromboplastin time (APTT) is an index of intrinsic clotting activity and prothrombin time (PT) can be used to evaluate the overall efficiency of extrinsic clotting pathway. An increscent APPT or PT indicates the deficiency in coagulation factors or the presence of coagulation inhibitors. Platelets are the main agents promoting hemostasis and blood clotting. When the maximum platelet aggregation rate (MPAR) increased, the blood viscosity became higher and more likely to cause thrombosis. Fibrinogen (Fbg) is one of the four commonly used clinical coagulation test indicators. An increase in Fbg is common in patients with angina pectoris, acute myocardial infarction and cerebral infarction diseases. The results were shown in Fig. 1. There were significant differences between control and model for all 10 indices. WBV at three shear rates significantly decreased in low molecular weight heparin (LMWH) group (P < 0.01) as well as DHI intermediate- and high-dose groups (P < 0.01). EAI, RCEI and ERI significantly decreased in LMWH group (P < 0.01) and DHI high-dose group (P < 0.01). LMWH significantly prolonged APTT and PT (P < 0.01) but DHI showed no significant effects at all dose levels. In DHI high-dose group, MPAR was suppressed and Fbg were significantly increased (P < 0.05), while LMWH had no significant effects on these two platelet aggregation indexes. In summary, the pretreatment of DHI could significantly reduce blood viscosity and downregulate blood coagulation in rats, thereby preventing the hypercoagulable state.

### Inhibitory effect of DHI on cerebrovascular thrombosis *in vivo*

**Figure 2:**
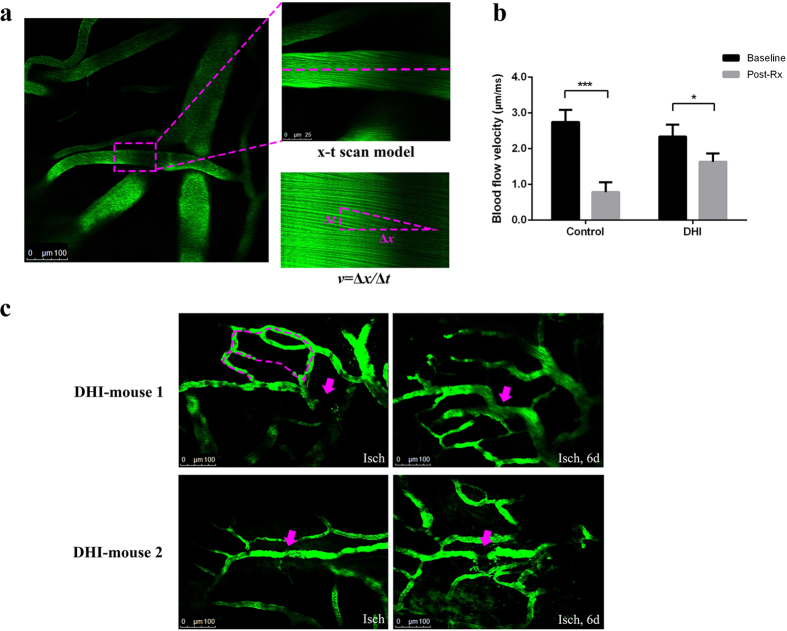
Effects of DHI on cerebrovascular thrombosis in mice. A cerebral microarteriole thrombosis mouse model was established by using the two photon laser scanning microscopy. (**a**) Measurement of arteriole blood flow-velocity by line-scans along the longitudinal axis of target arterioles. The slopes (△*x*/△*t*) of the measurement-angles are proportional to flow velocity. (**b**) Arteriole with diameter of 18 ± 3 μm and 3 ± 1 μm/ms in blood velocity was selected as the target vessel for occlusion. DHI was injected after the thrombus formation. After 30 min, the blood flow velocity was calculated to evaluate the effect of DHI. The graph depicts the mean ± standard deviation (n = 5). ^*^ P < 0.005 and ^***^ P < 0.0001 as compared with the baseline in each group. (**c**) New branches of arterioles appeared six days after the proximal vessels occluded in two mice of DHI group. Isch, after thrombus induction, before DHI treatment; Post Rx, after administration of DHI. Magenta dashed: location of disappeared vessels. Magenta arrows: occlusion site through laser irradiation.

Blood flow velocity was measured by two-photon laser scanning microscopy (TPLSM). The formation of thrombus in brain microarteriole of male C57BL/6 J mouse was induced by laser irradiation in bleach mode. The clots were firstly attached in the downstream vessel wall of injured area and finally adhered on the injured intimal layer after constant laser irradiation. The blood flow velocity of target arteriole was measured in x-t scan model through Leica software^24^ (Fig. 2a). In control group (see Supplementary Video S1), the blood flow velocity reduced significantly compared with that in baseline after thrombus formation (0.78 ± 0.28 vs 2.74 ± 0.34 μm/ms, P < 0.0001). While in DHI group (see Supplementary Video S2), the blood flow maintained at a relevant faster velocity after the clot occurred (1.63 ± 0.24 vs 2.34 ± 0.33 μm/ms, P = 0.0045, Fig. 2b). Furthermore, an interesting phenomenon was observed at some target arterioles after clot formation in the DHI group. New branches of arterioles appeared six days later after the proximal vessels occluded (Fig. 2c), which suggested that DHI might help to activate the growth of collateral vessel and alleviate the cerebral ischemia.

### Characterization of the chemical constituents in DHI

The identification of compounds in DHI was accomplished by UFLC-PDA-Triple TOF-MS/MS. The base peak chromatograms of DHI in positive and negative ion modes were shown in Supplementary Fig. S1. Multiple means were employed for identification and characterization of the constituents in DHI, such as reference standard comparison, quasi-molecular weight matching and MS/MS fragmentation pattern analysis. The accurate molecular weight of [M + H]^+^/[M + Na]^+^ in positive ion mode or [M-H]^−^/[M + HCOO]^−^ in negative ion mode with errors less than ± 5 ppm was qualified for the tentative identification. And then the final identity was made based on the MS/MS fragmentation. As a result, a total of 82 compounds, including 2 alkaloids, 3 nucleosides, 6 amino acids, 5 organic acids, 4 iridoid glycosides, 7 flavonoids, 5 quinochalcones, 39 phenolic acids, 8 tanshinones and 3 other compounds were identified or tentatively characterized in DHI. Among them, 19 peaks were unequivocally identified by comparing their retention time and MS data with those of authentic compounds. When there was no reference standard, the fragmentation behaviors of compounds with same basic skeleton obtained in this study or available data from literatures were taken into account. The compounds profile of ten batches of DHI commercial products were analyzed by UFLC-PDA-Triple TOF-MS/MS, and there was no significant difference in composition of ingredients as showing in Supplementary Table S1.

The attributions of the 82 identified compounds were also surveyed. As shown in Supplementary Table S1, 17 compounds were detected in both SM and CT herbs, which mainly included amino acids and nucleosides. This indicated that both of the two tested herbs contained a wide variety of nutritional components. Besides the common compounds, 32 compounds mainly consisted of phenolic acids and tanshinones were originated from SM, and 22 compounds included flavonoids, quinochalcones and iridoid glycosides were contributed by CT. Meanwhile, it is noteworthy that 11 phenolic acids in DHI were not detected in both herbs and they might be newly formed during DHI manufacturing process.

### Preparation of DHI prescription modified samples and UPLC fingerprints analysis

Uniform design was proposed by Fang and Wang based on the Quasi-Monte Carlo method or number-theoretic method^25^, which is capable of producing samples with high representativeness for multi-factor, multi-level experiments. In the present study, a two-factor, nine-level uniform design was adopted to insure differences among the nine DHI modified samples (see Supplementary Table S2).

**Figure 3:**
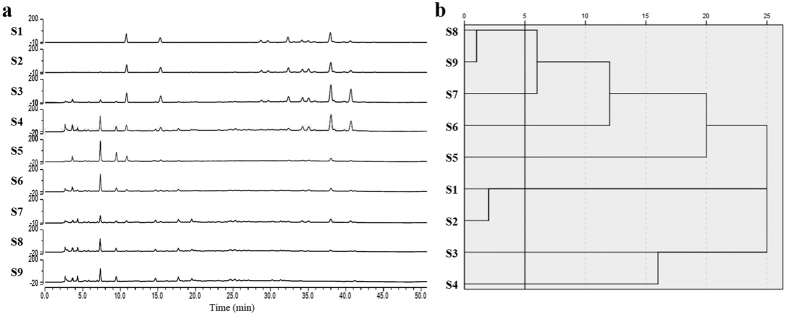
UPLC fingerprints analysis of nine DHI modified samples. (**a**) The UPLC fingerprints of nine DHI modified samples were detected at 288 nm wavelength. Thirteen main common peaks were identified based on the results of UFLC-PDA-Triple TOF-MS/MS analysis. (**b**) Cluster analysis results of DHI modified samples. Given the rescaled distance of 5, nine samples could be divided into 7 classes: S1 & S2, S3, S4, S5, S6, S7, S8 & S9.

In the UPLC fingerprints of DHI modified samples (Fig. 3a), 13 main common peaks were identified based on the results of UFLC-PDA-Triple TOF-MS/MS analysis, i.e., 5-hydroxymethylfurfural, danshensu, protocatechuic aldehyde, hydroxysafflor yellow A, p-coumaric acid, salvianolic acid H, salvianolic acid I, salvianolic acid D, methyl lithospermate, rosmarinic acid, lithospermic acid, salvianolic acid B and salvianolic acid A.

According to the peak areas of 13 selected components, 9 DHI modified samples could be divided into 7 categories: S1 and S2 belonged to a class, S8 and S9 belonged to a class, and the rest of the samples each represented a class (Fig. 3b), which indicated that there were chemical differences among the nine DHI modified samples and could be used for the next pharmacological experiments. The peak areas of these 13 components were adopted as independent component variables (see Supplementary Table S3).

### Effect of DHI modified samples on hemorheological properties and blood coagulation function in qi stagnation and blood stasis rats

**Figure 4:**
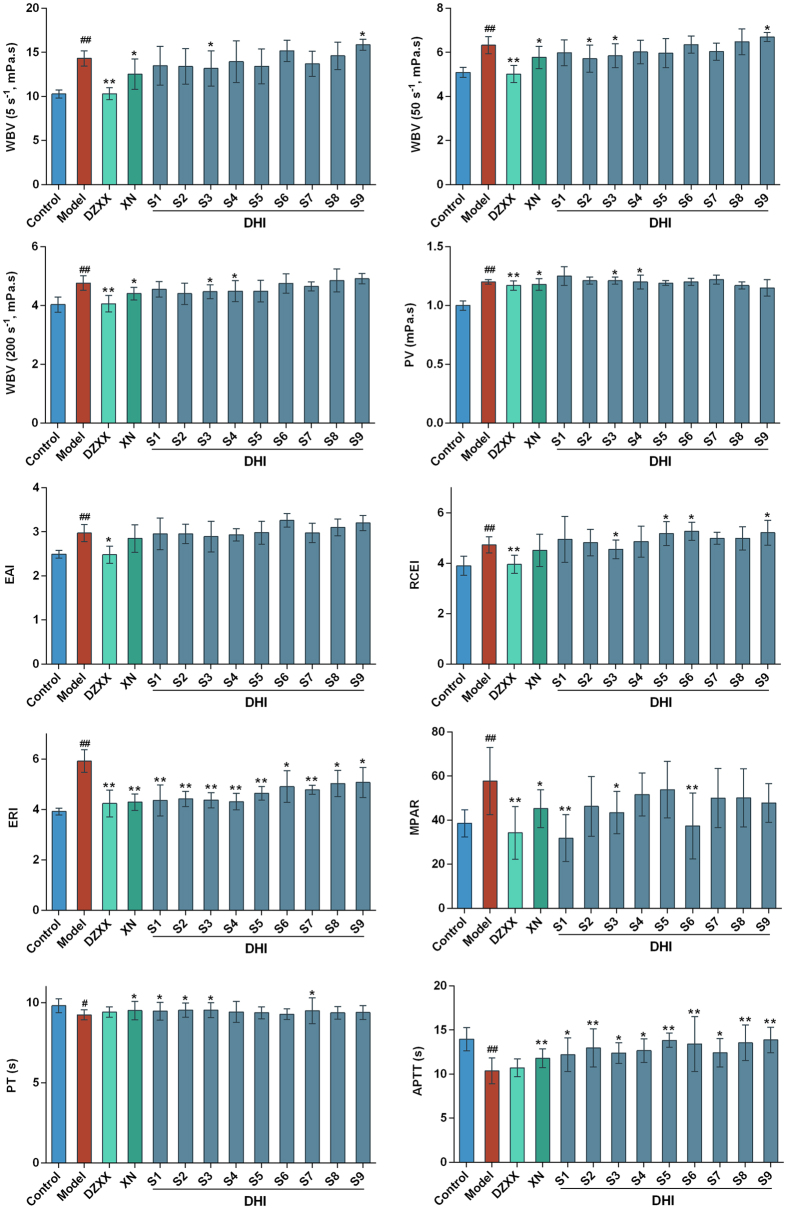
Effects of nine DHI modified samples on hemorheological properties and blood coagulation function in Qi stagnation and blood stasis rats. DZXX: Deng-zhan-xi-xin injection; XN: Xanthinol nicotinate injection; DHI: Dan-hong injection. Groups: control group, model group, DZXX group (4.4 ml/kg/d), XN group (3.2 mg/kg/d), and nine DHI modified samples groups S1–S9 (2.9 ml/kg/d). Control group and model group were injected with same volume of normal saline. Data are presented as mean ± standard deviation (n = 10). Pound signs indicate data that differed significantly from control (^#^P < 0.05, ^##^P < 0.01). Asterisks indicate data that differed significantly from model (*P < 0.05, **P < 0.01).

The rat model and test indexes were consistent with described above with a small modulation. Fbg was excluded because all tested drugs had little influence on it. And plasma viscosity (PV) was included for a more elaborate discussion on DHI’s effects on blood viscosity. Figure 4 showed the different effects observed in all 13 treatment groups, with particularly noticeable differences in 9 modified DHI groups. There were significant differences between the control rats and model rats for all pharmacological indices. WBV at all shear rates and PV increased significantly in model rats. The TCM injection Deng-zhan-xi-xin injection (DZXX) and the western medicine injection Xanthinol nicotinate injection (XN) were used as positive control. These two injections were both commonly used and have similar pharmacological effects of promoting blood circulation with DHI. After treatment, DZXX was significantly effective in decreasing WBV and PV. XN was significantly effective in reducing WBV (except WBV 30) and PV. For DHI samples, WBV and PV decreased significantly in S3 group. The red blood cell indices EAI, RCEI and ERI increased significantly in model rats. After treatment, DZXX was significantly effective in decreasing all the three RBC indices. XN was significantly effective in reducing ERI. DHI samples had less impact on EAI, however, ERI was significantly influenced in all DHI groups. RCEI decreased significantly in S3, S5, S6 and S9 groups. The coagulative parameters PT and APTT decreased significantly and MPAR increased significantly in model rats. After treatment, APTT was significantly influenced in all DHI groups. PT was prolonged in S1, S2, S3 and S7 groups. MPAR decreased significantly in S1, S3 and S6 groups. The results showed that indices differed greatly among the 9 DHI modified samples, and the possible reasons were discussed in subsequent analyses.

### Identification of bioactive components promoting blood circulation in DHI

A combination of different mathematical methods was adopted to comprehensively analyze the relationship between UPLC fingerprint data and pharmacological experimental data (see Supplementary Table S3 and Supplementary Table S4), including factor analysis, grey relational analysis, multiple linear regression analysis and radial basis function (RBF) neural network analysis.

The factor analysis simplifies multivariate data consisting of a large number of intercorrelated variables by grouping them into a smaller set of independent factors or clusters according to the basic underlying relationships between them. Factors are fewer in number than the original variables, account for a significant proportion of data variance, and are useful in predictive regression models. Five mutually independent factors were extracted from 10 intercorrelated pharmacological indexes and could reflect 97.47% of the original data information. The five factors each represented one aspect of the vascular system with clear clinical significance (see Supplementary Table S5): blood viscosity (F1), extrinsic clotting activity and RBC aggregation (F2), platelet aggregation (F3), intrinsic clotting activity and plasma proteins viscosity (F4), and RBC deformability (F5). The scores of F1-F5 were then calculated to be the new dependent variables F1–F5 (Table 1).

Grey relational analysis is suitable for solving problems with complicated interrelationships between multiple factors and variables and has been demonstrated as a useful approach to solve multiple-attribute decision-making problems^26^. Generally speaking, one of the main purposes of relation analysis is to identify the importance order of factors, so the grey relational degree (GRD) rankings are more significant. Regarded the factor F1-F5 as the reference sequence, and the 13 compounds as the independent variable sequence, the GRD results were calculated as to clarify the efficacy contribution of each compound. The results (Table 2) showed that P4, P1 and P5 were associated with F1 in relatively high degrees, which were greater than 0.7, suggesting that these three compounds were closely associated with the improvement of the blood viscosity. Compounds P2, P11 and P1 showed relatively high GRD with F2, which were between 0.6 to 0.7, suggesting that they played a role in improving the extrinsic clotting activity and RBC aggregation. P10, P6 and P2 had relatively high GRD with F3, which were greater than 0.7, indicating that they may possess strong pharmacodynamics on the inhibition of platelet aggregation. P10, P6 and P8 associated with F4 in relatively high degree, which were between 0.65–0.7, showing that they can be adjusted to some extent endogenous coagulation and plasma viscosity. They were all from SM. For factor F5, P13, P7 and P5 had GRD around 0.68, suggesting that they might make significant contributions on the improvement of RBC deformability.

**Figure 5:**
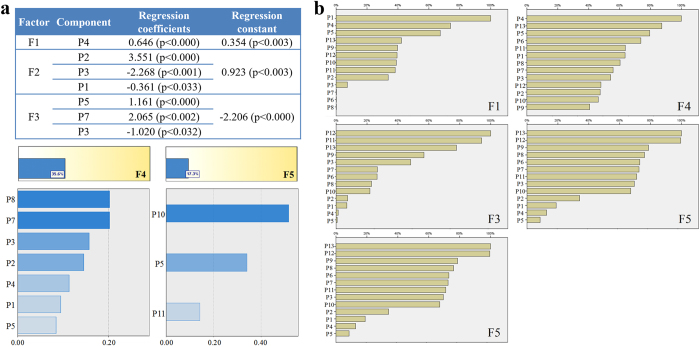
Relevance Analysis between five factors and 13 components. (**a**) Multiple linear regression results of F1, F2 and F3. For F4 and F5, since multiple linear regression model cannot be established (P > 0.05), the correlations were assessed by the relative importance results. (**b**) The relevance results between 13 components and five factors based on the radial basis function neural network analysis.

Multiple linear regression analysis was achieved by SPSS software. Factors F1-F5 were regarded as the dependent variables, and the fingerprint data of 13 compounds P1-P13 were regarded as the independent variables. When the linear regression model cannot be established (P > 0.05), the relative importance between dependent and independent variables was used to reflect the degree of association between the efficacy and compounds. Results were showed in Fig. 5a. For factor F1, only P4 was filtered into the equation, indicating that P4 had a significant impact on the improvement of blood viscosity, which was much higher than other compounds. For factor F2, components P2, P3 and P1 were filtered into the equation. P2 had a significant positive effect, while P3 and P1 showed negative impact on the exogenous coagulation and RBC aggregation. For factor F3, components P5, P7 and P3 were filtered into the equation. P5 and P7 showed significant positive effects, whereas P3 might play a negative role on the platelet aggregation effect. Factor F4 and F5 cannot establish accurate and reliable multiple linear equations (accuracy 35.6% and 17.3% respectively), thus the relative importance was used to evaluate the relation with the compounds. P8, P7 and P3 showed higher degree of importance than others on the endogenous clotting plasma viscosity, and they are all from SM. P10, P5 and P11 were significantly higher than the relative importance of the remaining two compounds, suggesting that P10, P5 and P11 may had more significant impact on the improvement of the RBC deformability.

RBF neural network analysis is one of the artificial learning methods that describe complex linear or non-linear relationships between inputs and outputs^27^. According to the RBF analysis results (Fig. 5b), P1, P4 and P5 showed much higher association degree with the factor F1, which were well consistent with the results of GRA, suggesting that they might have strong efficacy contribution on the improvement of blood viscosity. P11, P3 and P2 may play important roles in improving the exogenous coagulation and RBC aggregation which was corresponding to the factor F2. For factor F3, the importance degrees of P12, P11 and P13 were much higher than other compounds, suggesting they had strong influence on the platelet aggregation. For factor F4, components P4, P13 and P5 were closely associated with the effects on endogenous clotting and plasma viscosity. For factor F5, P13 and P12 were quite more important than other compounds, showing that both of them had significant impacts on the red blood cell deformability regulation.

**Figure 6:**
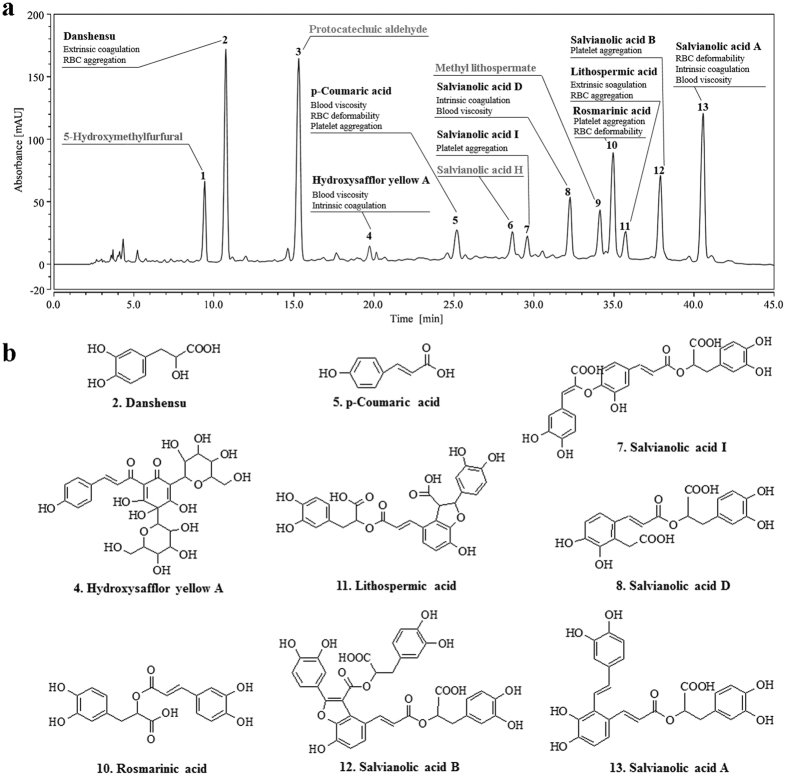
The standard bioactive fingerprint of DHI and its core bioactive components. (**a**) The UPLC fingerprint of DHI was detected at wavelength 288 nm. The core bioactive components are displayed in bold for qualitative or quantitative detection. (**b**) Structures of the core bioactive components promoting blood circulation in DHI.

Overall, grey relational analysis, multiple linear regression analysis and RBF network analysis were combined to control the mutual interference among the 13 component variables to better assess the data. Through comprehensive analysis, we determined that danshensu, hydroxysafflor yellow A, p-coumaric acid, salvianolic acid I, salvianolic acid D, lithospermic acid, rosmarinic acid, salvianolic acid B and salvianolic acid A could serve as the core bioactive components of DHI and that they acted on different aspects of the vascular system. Thereby the bioactive UPLC fingerprint of DHI (Fig. 6) was established based on its core bioactive components.

### Validation of the blood circulation promoting effects of DHI core bioactive components (CBC) by qi stagnation blood stasis rats model

**Figure 7:**
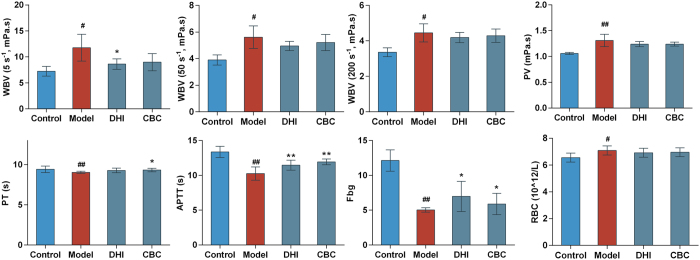
Effects of DHI and its core bioactive components on hemorheological properties and blood coagulation function in Qi stagnation and blood stasis rats. DHI: Dan-hong injection, CBC: Core bioactive components of DHI. Groups: control group, model group, DHI group (2.9 ml/kg/d) and CBC group (2.9 ml/kg/d). Control group and model group were injected with same volume of normal saline. Data are presented as mean ± standard deviation (n = 8). Pound signs indicate data that differed significantly from control (^#^ P < 0.05, ^##^P < 0.01). Asterisks indicate data that differed significantly from model (*P < 0.05, **P < 0.01).

The contents of each compound in CBC sample were consistent with that in DHI sample. First of all, the contents of components in DHI sample were determine by UPLC. The quantitative method was same as published previously and the contents were calculated based on regression equations of 8 components (see Supplementary Table S6 and Supplementary Table S7). The *in vivo* experimental validation results (Fig. 7) showed that DHI and its core bioactive components both could significantly alleviate the circulation dysfunction, especially possessed similar effects on reducing blood viscosity and improve the function of blood coagulation. The efficacy of DHI was slightly better than its core bioactive components but showed no significant difference.

### Comparisons of permeation of main compounds across the blood-brain barrier after administration of DHI and SM single herb injection to rats

**Figure 8:**
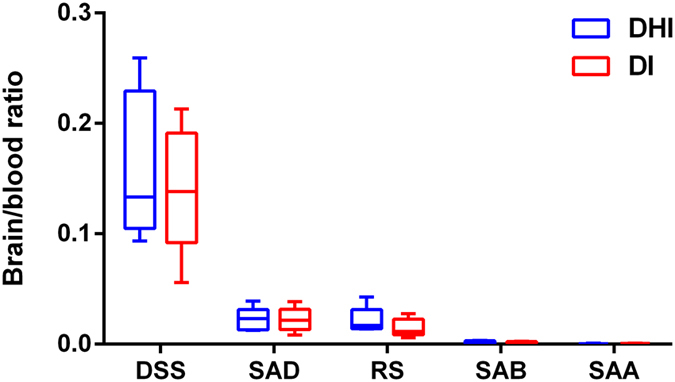
The brain/blood ratio of five main compounds 5 min after intravenous injection of DHI and single herb Danshen injection in rats (n = 5). DHI: Dan-hong injection, DI: single herb SM injection. DHI and DI were prepared with the same batch of SM herb. DSS: Danshensu, SAD: Salvianolic acid D, RS: Rosmarinic acid, SAB: Salvianolic acid B, SAA: Salvianolic acid A. For each box plot, the middle line indicates the median, the box indicates the range of the 25th to 75th percentiles of the total data, and the whiskers are from minimum to maximum.

Another evident help understand the scientificity of TCM prescription composition rules is from a comparison of the brain/plasma ratios of some main compounds when rats were administrated SW with or without CT. According to the results aforementioned, DHI could not only inhibit the thrombus formation and maintain the blood flow stability to some extent, but activate the growth of collateral vessel. In order to exert the therapeutic action of DHI, some potential molecules needed to penetrate the blood-brain barrier (BBB) and stay in the brain tissue. Meanwhile, the levels of ingredients from CT were too low to detected in rat plasma, so whether CT will have any impact on the composition of SM in brain. The brain/blood ratio was calculated by dividing the concentration of compound in each gram of mouse brain by the concentration in one ml of blood. The compounds detected in the brain tissue include danshensu, salvianolic acid D, rosmarinic acid, salvianolic acid B, salvianolic acid A. As showed in Fig. 8, danshensu, salvianolic acid D, rosmarinic acid generated higher brain/blood ratios than salvianolic B and salvianolic A. When SM was used with CT, notable increasing trends in the blood/brain ratio of danshensu and rosmarinic acid can be observed. While salvianolic acid D did not show much difference. Thus the presence of CT might facilitate some compounds of SM penetrating the blood-brain barrier.

## Discussion

A pattern or syndrome that reflects multi-system changes is actually the target of TCM treatment. Therefore, the establishment of whole animal model that reflects certain functional state or certain syndrome is of great importance in the pharmacological evaluation of TCM. According to TCM theories, the pathological basis of coronary heart disease mainly includes qi stagnation, blood stasis, endogenous accumulation of phlegm turbid and heart meridian obstruction. The rat model used in this study was a simulation of the qi stagnation and blood stasis state in patient. Based on TCM principle that cold stimulation could block qi movement, ice water exposure was used to mimic the qi stagnation state in rat. Qi stagnation could cause blood stasis, and the maladjustment was aggravated when coupled with Adr’s effects on increasing heart rate, force of heart contraction and skin vasoconstriction. We also speculate that qi in TCM theory may be related to the energy metabolism of the organism.

The results showed that the anticoagulants LMWH obtained better thrombolytic activity than DHI at clinical equivalent dose, while no significant differences were observed between LMWH and DHI intermediate-/high-dose groups. The effect of LMWH on APTT was superior to that of DHI high dose, while DHI had advantages on the improvement of Fbg. Compared to the therapeutic effect of LMWH which was of high pertinency, the effects of DHI possesses characteristic of gentle but multi-aspects. Nevertheless, there are some differences in the clinical indications of LMWH and DHI, the former are aimed at acute symptoms and usually only used for short term, whereas the latter is good at chronic disease treatment and suitable for long-term use. DHI especially for elderly patients with cardiovascular and cerebrovascular disease efficacy and rarely appear easy to hemorrhage risk situation. DHI produces a definite curative effect on cardiovascular and cerebrovascular disease in elderly patients and rarely lead to bleeding tendency.

Thrombus model of cerebral arterioles was developed in C57BL/6 J mouse with laser irradiation of bleach model. The mechanisms of clot formation induced by laser irradiation had been demonstrated to be the primary endothelial and basement membrane injuries^24^. The formation steps of such thrombosis could be similarly described by platelet functions and coagulation cascade at a functional level^28^, combined with platelets and fibrin. In this study, the arteriole within limited range of vessel diameter and flow velocity was selected to develop thrombus by a depth-dependent laser irradiation. The results suggested that DHI could inhibit the thrombus formation and maintain the blood flow volume to some degree. In addition, the occurrence of branches of arterioles in the 6d-obsevation suggested that in ischemia condition, DHI might help to activate the growth of collateral vessels and enhance the collateral circulation, which indicated that the pharmaceutical effects of DHI include not only the regulation of several crucial targets and pathways, more importantly, the modulation of other associated general changes that promote healing process.

Since TCM is multi-components mixtures, the systematic identification of chemical constituents is thus of great importance, not only for providing scientific evidence for the quality control, but also for the clarification of pharmacodynamic basis and the development of new drugs. In the present study, 82 compounds were identified in DHI by UFLC-PDA-Triple TOF-MS/MS. In addition to the 71 identified constituents from SM or CT, 11 compounds were found to be newly formed during the manufacturing process. Polyphenolic acids such as salvianolic acid B were found to be unstable during some essential production procedures of SM preparations, including heat extraction and adjustment of the pH value^29^. For instance, in the autoclaved sample of salvianolic acid B, 12 peaks were detected and identified as caffeic acid, danshensu, protocatechuic aldehyde, salvianolic acid F, lithospermic acid, prolithospermic acid, salvianolic acid D, salvianolic acid E, rosmarinic acid, salvianolic acid A, salvianolic acid B and isosalvianolic acid B by HPLC-TOF-MS^30^. Consequently, the contents of these compounds in the finished products may vary between batches. The ingredients formed during the preparation process are also probably important to the effectiveness and safety of Chinese herbal medicine^31^. Therefore, the dynamic changes of chemical constituents during the manufacturing process should be thoroughly studied and taken into consideration when setting standards, so as to keep the consistency of chemical constituents in the decoction and the final products.

When a single constituent was isolated from TCM and tested, it usually cannot achieve the expected activity and even can be toxic^1^,^5^. What’s more, the concentration of single compound in TCM is usually not sufficient to exert certain pharmacological activities. There may be synergistic and/or additive actions between different compounds via targeting same or diverse targets in the same pathological pathways. From this consideration, this study takes a holistic approach to examine the relationship between chemical constituents and therapeutic effects of DHI on promoting blood circulation, which is a major mechanism in explaining the clinical benefits of DHI to cardiovascular diseases.

It was found that the two compounds from CT, hydroxysafflor yellow A and p-coumaric acid were highly relevant to the regulation of blood viscosity. Danshensu and lithospermic acid were relevant to extrinsic clotting activity and RBC aggregation. Salvianolic acid I, salvianolic acid B, rosmarinic acid and p-coumaric acid were relevant to platelet aggregation. Hydroxysafflor yellow A, salvianolic acid D and salvianolic acid A were relevant to intrinsic clotting activity and plasma proteins. Salvianolic acid A, rosmarinic acid and p-coumaric acid were relevant to RBC deformability. Besides, according to the attribution of 9 bioactive components, CT showed strong influence on the viscosity of blood and plasma. And SM significantly affected the extrinsic coagulation system and intrinsic coagulation activity. The results also showed that 5-hydroxymethylfurfural and protocatechuic aldehyde might have negative effects on extrinsic clotting activity, RBC aggregation and platelet aggregation (Fig. 5a). 5-Hydroxymethylfurfural is a common product of the Maillard reaction and induction of aberrant colonic crypt foci had been reported^32^. Therefore, the limitation test of 5-hydroxymethylfurfural should be considered in the quality control of DHI. Studies have shown protocatechuic aldehyde to possess beneficial antioxidant, anti-inflammatory and antifibrogenic effects^33^,^34^,^35^. What’s more, in cardiotonic pill, protocatechuic aldehyde was considered to be the leading activity contributor for inhibition of NO production in lipopolysaccharide (LPS)-stimulated macrophages^5^. Hence, protocatechuic aldehyde may play a role in the treatment of cardiovascular disease through antioxidant and anti-inflammatory pathways instead of immediately affecting blood circulation. Although the blood circulation promoting activities of these compounds have been previously reported^36^,^37^,^38^, this study elucidated the exact contribution of each component to the whole activity of DHI for the first time.

Among the nine DHI modified samples, S3 is prepared according to the original formula ratio and showed the most prominent effectiveness, which demonstrated that SW and CT herbs might exert the overall efficacy through the interaction and the compatibility proportion was reasonable and effective. The scientificity of SW and CT compatibility also lied in the facilitation of the penetration of SW main compounds into brain tissue. Nevertheless, CT may have a risk of leading to increased bleeding tendency^39^, but DHI has a very good safety record which the incidence rate of adverse drug reactions was 3.50‰^16^. This indicated that SW and CT in combination possibly could reduce the bleeding tendency caused by CT.

Meanwhile, the detached information of the chemical fingerprint and the pharmacological effects is still a crucial question that we need to pay attention in order to guarantee the consistency of quality^40^,^41^,^42^. Thus, the identified bioactive compounds could be further associated to the chemical fingerprint, as well as elucidated the relationship between TCM chemical profile and therapeutic effects, which is meaningful to the quality control of TCM.

Both TCM and organism are complex systems and the specific interactions between them are extremely complicate. For instance, this study demonstrated that one specific aspect of the vascular system might be affected by two or more components, and the same component might have a positive effect on one aspect yet a negative effect on another, which reflects both the synergistic and the antagonistic actions. Therefore, the pharmacological mechanisms of the core components identified in this study are needed to be further explored and verified. Unlike isolating and screening for single bioactive compound, this study focuses on the integrity and internal relations of TCM. The results may provide basis for the scientific explanation of the clinical benefits of DHI. And the approaches in this study could also be widely applied to the comprehensive evaluation of other TCM.

## Methods

### Materials and reagents

Ten batches (nos 13111002, 13111003, 13112001, 13112002, 13112003, 13112004, 13112005, 13112006, 13112007 and 13112008) of DHI and its raw material herbs *Salvia Miltiorrhiza* (batch no. 141103) and *Carthamus tinctorious* (batch no. 141101) were provided by Buchang Pharmaceutical Co., Ltd. (Shandong, China). The plant species were identified by Prof. Wenbo Liao from Sun Yat-Sen University. The voucher specimens were deposited in our laboratory. Deng-zhan-xi-xin injection (DZXX, batch no. 20131240) was obtained from Yunnan Biovalley Dengzhanhua Pharmaceutical Co. Ltd. (Yunnan, China). Xanthinol nicotinate injection (XN, batch no. 20131101) was from Polifarma (Nanjing) Co., Ltd. (Jiangsu, China). Sodium chloride injection (normal saline, SCI, 0.9%, batch no. XB140210) was obtained from JiangXi Kelun Pharmaceutical Co. (Jiangxi, China). Fluorescein isothiocyanate-dextran (FITC, 2000 KD, Lot no. SLBB6384V) was purchased from Sigma (Germany) and was dissolved in SCI with a concentration of 5% for imaging under TPLSM.

The authentic standards of adenine, uridine, succinic acid, isoleucine, adenosine, phenylalanine, danshensu, protocatechuic acid, vanillic acid, tryptophane, protocatechualdehyde, hydroxysafflor yellow A, caffeic acid, p-coumaric acid, rosmarinic acid and salvianolic acid B were purchased from the National Institute for the Control of Pharmaceutical and Biological Products (Beijing, China). Salvianolic acid D, lithospermic acid and salvianolic acid A were purchased from Shanghai YuanMu Biological Technology Co. Ltd. (Shanghai, China) with purity > 98% detected by high performance liquid chromatography.

Acetonitrile of LC-MS grade was purchased from Fisher Scientific (Pittsburgh, PA, USA). All water used was distilled and further purified by a Milli-Q system (Millipore, Milford, MA, USA). Other reagents used in the experiment were of analytical grade.

### Animals

All animal experiments in this study were approved by Animal Ethics Committee of the School of Life Sciences, Sun Yat-sen University (permission No. 20142000226) and were performed in accordance with the with institutional guidelines for animal experiments. Sprague-dawley (SD) rats and C57BL/6 J mice were purchased from Guangdong medical experimental animal center (certification No: SCXK-(Yue) 2013-0002) and raised in the specific-pathogen free (SPF) laboratory of ocean and traditional Chinese medicine laboratory of Sun Yat-sen University (license: SYXK-(Yue) 2014-0020). The temperature of the SPF laboratory was kept at 20–23 °C and the relative humidity was 50–65%. Animals were kept under 12-h dark-light cycle and fed by standard laboratory pellet feed. The harm to animals was reduced to minimal extent during experimental process by taking appropriate measures.

### Pharmaceutical evaluation of DHI

The effects of DHI on hemorheology and blood coagulation function were evaluated in a rat model of blood stasis and qi stagnation^21^. Sixty male SD rats weighed 220–240 g were randomly divided into 6 groups with 10 rats in each group: control, model, LMWH (50 μg/kg/d), DHI low dose (0.8 ml/kg/d), DHI intermediate dose (1.5 ml/kg/d) and DHI high dose (3.0 ml/kg/d). Rats received treatment via intramuscular injection once a day for 10 consecutive days. Control and model groups were injected with same volume of normal saline. Modeling was performed thirty minutes after the last administration. All rats except control group were subcutaneously injected with Adr (0.8 mg/kg), and the control group was subcutaneously injected with same volume of normal saline. Two hours later, except control group, all rats were kept in a water-ice bath (0–4 °C) for 5 min. And after another 2 h Adr (0.8 mg/kg) was subcutaneously injected again except that control group injected with normal saline. All rats were fasted for 12 h after the re-injection and then were given the last administration. Thirty minutes after the last administration, rats were anesthetized by intraperitoneal injection of chloral hydrate (10%, 3.5 ml/kg). Then blood was collected from the abdominal aorta into plastic tubes contained 3.8% sodium citrate (citrate/blood: 1/9, v/v). All the blood samples, used for the detection of efficacy indicators related to hemorheology, platelet aggregation and blood coagulation activity, were processed and examined strictly by standard operating procedures. APTT and PT were detected by full-automatic blood coagulation analyzer (Sysmex, CA-510). EAI, RCEI, ERI and WBV at 5/s, 50/s and 200/s shear rate were detected by full-automatic self-cleaning hemorheology analyzer (Beijing Precil, LBY-N6B). MPAR and Fbg were detected by Platelet aggregation analyzer (Beijing Precil, LBY-NJ4).

### Microarteriole thrombus induced by laser irradiation in mouse model *in vivo*

Ten male C57BL/6 J mice (20–24 g, 8 weeks) were divided into two groups of five mice each: control group and DHI group (5.0 mL/kg). The mouse was anesthetized with chloral hydrate (4.2%, 10.0 mL/kg) and placed on the heating pad to keep warm at 37.0 °C. A 1.0 cm incision was made on the midline of scalp from between the ears to the eyes. The thin periosteum on the skull was removed slightly to expose the bregma clearly. A 2 × 2 mm^2^ cranial window was produced over the left or right parietal cortex and grinded with a micro-drill^43^. The whole procedure was supposed to be careful, avoiding internal bleeding, in order to acquire a thin and clear cranial window. After the surgery, a metal plate was glued at the edge of the cranial window. The mouse was injected with FITC (10.0 mL/kg) by tail vein and fixed on the stage of TPLSM (Leica DM6000, Germany). After the experiment, the mouse was sutured up and raising for the next observation.

The image of cerebral vessel throughout the cranial window was first obtained with a 10× magnification air objective and transferred to a water-immersion one (40× magnification) for high-resolution. The arteriole with diameter of 18 ± 3 μm and 3 ± 1 μm/ms in blood velocity was selected as the target vessel for occlusion. Target arteriole was them place in the center of the field and zoomed in at 20 times magnification. A bleach model was used to induce endothelium injures by irradiating the vessel wall within the lumen. The laser was set at 800 nm and started irradiating with energy setting at depth-dependence (Max. power 3.5 W). The modeling irradiation was continued until a clot was visualized and the blood flow slowed down.

After the thrombus occurred, the mouse in DHI group was drugged with DHI intraperitoneally while the model group was injected with SCI in the same volume. Thirty minutes later the target arteriole was observed again to record the occlusion situation in order to evaluate the effect of DHI.

The blood flow velocity of target arteriole was calculated and presented as mean ± standard deviation (n = 5) by Leica Las AF (version 2.6.0) with results exported through GraphPad Prism (GraphPad Software, Inc., version 6.01).

### Identification of chemical constituents in DHI by UFLC-PDA-Triple TOF-MS/MS

The mixed solution of 19 reference standards for identification was prepared at the concentration of ~0.1 μg/mL for each compound in methanol. An aliquot of 2 mL DHI sample was accurately diluted to a 10 mL volumetric flask with acetonitrile-0.1% formic acid solution (2:98, v/v). The grounded SM and CT herbs 0.5 g were separately ultrasonic extracted with 50 mL 75% methanol for 50 min at 40 kHz. All samples were filtered through a 0.22 μm membrane filter before injection.

A Shimadzu UFLC system (Shimadzu Corp., Kyoto, Japan) equipped with a LC-20AD-XR binary pump, an SIL-20AD-XR autosampler, a CTO-20A column oven and a SPD-M20A Photo-Diode Array detector was coupled to an AB SCIEX Triple TOF-MS 5600^+^ system (AB Sciex Foster City, CA) via an electrospray ionization (ESI) source. Chromatographic separation was carried out on a Phenomenex C_18_ column (2.1 mm × 100 mm, 2.6 μm) at 40 °C. The mobile phase consisted of 0.1% formic acid acetonitrile (A) and 0.1% aqueous formic acid solution (v/v) (B). In order to achieve fast and comprehensively identification, the gradient elution program was 2% to 40% A at 0–20 min, and 40% to 98% A at 20–25 min. The injected volume was 5 μL with the flow rate kept at 0.3 mL/min. UV spectra were obtained by scanning from 190 to 400 nm. Both positive and negative-ion mass spectra were recorded over the range m/z 100–1500. Nitrogen was used as nebulizer and auxiliary gas. The conditions of ESI source were as follow: ion source gas1 55 psi; ion source gas2 55 psi; curtain gas 35 psi; temperature 550 °C; ion spray voltage floating 5500 V; collision energy 45 V; collision energy spread 20 V; declustering potential 60 V. The UFLC-PDA-Triple TOF-MS/MS data was analyzed using PeakView software (AB SCIEX, Foster City, CA). Peak identifications were based on comparison of retention times, quasi-molecular weights and MS/MS spectra with standard references and the known compounds in SM and CT herbs.

### Preparation of DHI prescription modified samples

Based on the original DHI formula ratio, the prescription modified samples were prepared by changing the content of SM and CT. The specific weights of the 2 herbs were designed according to a two-factor, nine-level uniform design. Sample S3 was the original formula. For each sample, the two herbs were decocted together twice with water and the decoctions were mixed and concentrated. Then ethanol was added and refrigerated. The supernatant was later filtered followed by the recovery of ethanol. The residue was redissolved with water for injection. Then activated carbon was added, boiled and filtered. After adjusting the volume with water for injection and adjusting pH to 6.5 with NaOH solution, the filtrate was potted and sterilized.

### UPLC fingerprints analysis

DHI modified samples were diluted to 1:5 v/v with 0.5% aqueous formic acid, and then filtered through a 0.22 μm membrane filter before injection. UPLC analyses were carried out by using a Dionex Ultimate 3000 series UPLC system coupled with a diode array detector (DAD). The chromatographic fingerprints were obtained from a Welch Ultimate XB-C_18_ column (4.6 mm × 250 mm, 5 μm). The gradient elution was performed as previously described by using acetonitrile (A) and water with 0.5% formic acid (B) at the flow rate of 1 mL/min^44^. The program was as follows: 0–15 min, 2–10% (A); 15–20 min, 10–17% (A); 20–45 min, 17–28% (A); 45–48 min, 28–60% (A). An aliquot of 5 μL of each sample solution was injected into the UPLC–DAD system and detected at 288 nm. Peak areas were calculated with a Chromeleon 6.8 Chromatography Data System (Dionex) and cluster analysis of the nine DHI samples was conducted in SPSS 18.0 based on the peak areas. The clustering method was between-groups linkage, and the distance calculating method was Pearson’s correlation.

### Pharmacodynamic evaluation of DHI modified samples

One hundred and thirty male SD rats weighed 220–240 g were randomly divided into 13 groups with 10 rats in each group: control, model, XN (3.2 mg/kg/d), DZXX (4.4 ml/kg/d), and 1–9 DHI modified samples (2.9 ml/kg/d). Rats received treatment via intramuscular injection once a day for 10 consecutive days. Control and model groups were injected with same volume of normal saline. The rat model was same as described above. The indexes detected were also the same but with a slight modification where Fbg was replaced with PV. And PV was detected by full-automatic blood coagulation analyzer (Sysmex, CA-510). Data were documented as mean ± standard deviation. SPSS 18.0 software was used to carry out statistical analyses. One-way analysis of variance, student’s t-test and Dunnett’s multiple comparisons were used for comparing the results among groups. P-value less than 0.05 or 0.01 represented statistical significance.

### Relevance analysis between UPLC fingerprints and drug effects

The areas of the main peaks were regarded as independent component variables and were divided by each corresponding average area in 9 DHI samples for nondimensionalization. The average values of each pharmacological indexes were calculated for each group. Where the values showed negative correlation with the effects (the larger the value, the weaker the effect), they were converted by taking the reciprocal. Then the same nondimensionalization procedure was conducted by dividing each value by the corresponding average in 9 DHI modified sample groups.

Factor analysis was first used to reduce the high dimensionality of variable space, and five factors with no mutual correlation were extracted from 10 intercorrelated indices. The relevance between 10 indices and 5 factors was calculated with a rotation matrix method to explain the clinical meaning of the factors. The scores of five factors were then calculated to be the new dependent variables F1-F5. The processes above were implemented in SPSS 18.0 (Factor Analysis, Data Reduction, Analyze).

In grey relational analysis, the grey relational grade (GRD) is used to rank the influence of comparative series, which can be represented by the relative distance between the comparative series and reference series in an imaging grey space without making any prior assumptions about the distribution type. In order to calculate GRD, three steps were carried out, including grey relational generation, calculation of grey relational coefficient and calculation of the GRD. Grey relational generation consists of defining of data series and standardization of the original data. In this study, the original data has been nondimensionalized earlier. The reference series (x_0_) denotes five factors (the scores of F1-F5 of nine DHI samples), and comparative series (x_i_) are described as 13 component variables (dimensionless data of peak areas of P1-P13). The grey correlation coefficient (ξi(k)) between the reference sequence x_0_(k) and comparative sequence x_i_(k) is calculated using equation (1):


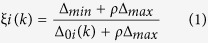


where Δ_0i_(k) is the deviation series of reference series x_0_(k) and comparative sequence x_i_(k), and equation (3) is applied to calculate the maximum and minimum deviations. The value of distinguish coefficient ρ is ranging from 0 to 1. In this study, the value of ρ was considered as 0.5.


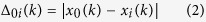



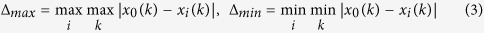


GRD shows the relationship among the series and is calculated using equation (4):


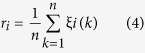


where n is the number of performance characteristics. The higher the GRD, the greater the effect of the corresponding component.

For multiple linear regression analysis, the multiple linear regression equations were constructed between the five factors F1-F5 (dependent variables) and DHI components P1-P13 (independent variables). The applied regression model was as equation (5).


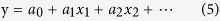


where y represents each factor; x_1_ and x_2_ represent the peak areas of the components; a_0_ is the regression constant; a_1_ and a_2_ are the regression coefficients for each component.

Once a strict regression equation was established (P < 0.05), the regression coefficients could be used to evaluate the contribution of each component. In case of no available equations (P > 0.05), the relative importance between dependent variables and independent variables were directly calculated. The processes above were implemented in SPSS 18.0 (Automatic linear model, Regression, Analyze).

In RBF analysis, the independent variables components P1-P13 were selected as input vectors and were propagated to the hidden layer for training basis function centers. The distance between the input vector and its own center was calculated and then transformed using the basis function. The output of each node in the hidden layer is multiplied by its connection weight and then fed into the output layer. The output layer consisted of dependent variables factors F1-F5, which were the linear addition of all the outputs of the hidden layer. Thereby, the RBF network between the components P1-P13 variables and each factor (F1-F5) was constructed. And the connection weight of variable was calculated and was used to evaluate the contributions of each component. All the processes above were implemented in SPSS 18.0 (RBF, Neural Networks, Analyze).

### Evaluation of the blood circulation promoting effects of the core bioactive components of DHI

This part of study was designed to experimentally validated the calculated core bioactive components of DHI by the rat model of blood stasis described above. Firstly, the contents of DHI core bioactive components were analyzed using an external standard method by UPLC. The column was Elite Hypersil ODS2 (4.6 × 250 mm, 5 μm) and other analysis conditions were same as the UPLC fingerprints analysis. The mixed standard solutions of danshensu, hydroxysafflor yellow A, p-coumaric acid, salvianolic acid D, rosmarinic acid, lithospermic acid, salvianolic acid B and salvianolic acid A were prepared by dissolving accurately weighed standards in acetonitrile-0.5% formic acid solution (50:50, v/v) to yield the concentrations of 783.6, 130.4, 39.40, 432.2, 227.1, 98.98, 721.5, and 366.9 μg/mL, respectively, for their various concentrations in DHI. A series of working solutions of appropriate concentrations was obtained by diluting the mixed standard solution. The preparation method of DHI (batch no. 15121018, Buchang Pharmaceutical Co., Ltd., Shandong, China) were same as described above. Then according to the content of each component in DHI, a normal saline solution contains the same amount of danshensu, hydroxysafflor yellow A, p-coumaric acid, salvianolic acid D, rosmarinic acid, lithospermic acid, Salvianolic acid B and salvianolic acid A was prepared as the core bioactive components sample.

Thirty-two SPF SD rats, weighed 220–240 g, male only, were obtained from Guangdong medical experimental animal center (certification no: SCXK-(Yue) 2013–0002) and raised in SPF laboratory of ocean and traditional Chinese medicine laboratory of Sun Yat-sen University (license: SCXK-(Yue) 2014-0020). Rats were divided randomly into 4 groups: control, model, DHI (2.9 ml/kg/d), Core bioactive components (CBC 2.9 ml/kg/d) with 8 rats in each group. The blood stasis rats modelling method and detection method were the same as described above.

### Determination of the brain/blood ratio of main compounds in rats after administration of DHI and SM single herb injection (DI)

Ten SD rats weighed 220–240 g were divided randomly into 2 groups: DHI and DI with 5 rats in each group. DHI and DI samples used were the DHI modified sample S3 and S1. Plasma concentration and brain accumulation were determined 5 min after intravenous injection with DHI or DI (2.9 ml/kg). The UFLC-PDA-Triple TOF-MS/MS detection method was same as described above.

## Additional Information

**How to cite this article:** Li, P. *et al*. Toward a scientific understanding of the effectiveness, material basis and prescription compatibility of a Chinese herbal formula Dan-hong injection. *Sci. Rep.* **7**, 46266; doi: 10.1038/srep46266 (2017).

**Publisher's note:** Springer Nature remains neutral with regard to jurisdictional claims in published maps and institutional affiliations.

## Supplementary Material

Supplementary Information

Supplementary Vedio S1

Supplementary Vedio S2

## Figures and Tables

**Table 1:** The scores of five factors of nine DHI modified samples.

Samples	F1	F2	F3	F4	F5
S1	0.31	0.03	1.98	0.81	0.02
S2	0.37	1.77	0.18	−0.33	0.53
S3	1.17	0.76	0.13	−1.11	−0.94
S4	0.44	−0.07	−0.79	0.77	2.12
S5	1.37	−1.43	−1.01	−0.47	−0.42
S6	−0.54	−1.48	1.17	−0.36	0.24
S7	−0.34	0.21	−0.65	1.93	−1.44
S8	−1.15	0.08	−0.71	−0.07	0.04
S9	−1.62	0.13	−0.31	−1.15	−0.14

F1-F5 represented Factor 1–5 respectively. S1–S9 represented the nine DHI modified samples. The scores are used as new dependent variables for the subsequent calculations.

**Table 2:** The grey relational analysis results between 13 components and five factors.

Factors	P1	P2	P3	P4	P5	P6	P7	P8	P9	P10	P11	P12	P13
F1	0.7524	0.6589	0.6222	0.7945	0.7217	0.6354	0.6174	0.6147	0.6547	0.6607	0.6325	0.6183	0.6411
F2	0.6370	0.6645	0.6260	0.5909	0.5818	0.6288	0.6281	0.6278	0.6197	0.6083	0.6406	0.6352	0.6117
F3	0.6499	0.7318	0.7162	0.6606	0.6686	0.7318	0.7193	0.7266	0.7266	0.7446	0.7178	0.7273	0.7252
F4	0.6412	0.6409	0.6544	0.6067	0.5841	0.6704	0.6569	0.6593	0.6428	0.6891	0.6269	0.6409	0.6291
F5	0.6387	0.6706	0.6738	0.6518	0.6837	0.6788	0.6860	0.6830	0.6831	0.6624	0.6804	0.6687	0.6879

The larger the value, the higher the relevance between components and factors.
