# Supplementary material for: Toward a scientific understanding of the effectiveness, material basis and prescription compatibility of a Chinese herbal formula Dan-hong injection
Source: Sci Rep. 2017 Apr 10;7:46266. doi: 10.1038/srep46266 (PMC5385876; doi:10.1038/srep46266)
Supplement: Supplementary Information [file srep46266-s1.pdf]

Supplementary Information

Manuscript title:

Toward a scientific understanding of the effectiveness, material basis and prescription compatibility of a Chinese herbal formula Dan-hong injection

Authors:

Panlin Li, Weiwei Su, Sha Yun, Yiqiu Liao, Yinyin Liao, Hong Liu, Peibo Li, Yonggang Wang, Wei Peng, Hongliang Yang

Guangdong Engineering and Technology Research Center for Quality and Efficacy Re-evaluation of Post-marketed TCM, Guangdong Key Laboratory of Plant Resources, School of Life Sciences, Sun Yat-sen University, Guangzhou 510275, P.R. China

Correspondence and requests for materials should be addressed to H.L.Y. (email: yaohliang@mail.sysu.edu.cn)

Supplementary Table S1. Identification of the chemical constituents of DHI by UFLC-PDA-Triple TOF-MS/MS.

NO	Formula	T_R (min)	[M+H]+ (error)	$[M-H]^-$ (error)	Fragment ions in positive (+) ion mode[a]	Fragment ions in negative (−) ion mode	Identification	Attribution
1	$C_5H_9NO_2$	0.90	116.0706 (-0.3)	-	70.0673$[M+H-HCOOH]^+$	-	Proline	SM/CT
2	$C_5H_5N_5$	1.03	136.0618 (0.2)	134.0471 (-0.9)	119.0358$[M+H-NH_3]^+$, 109.0503, 94.0409, 92.0255$[M+H-NH_3-HCN]^+$, 65.0160$[M+H-NH_3-2HCN]^+$, 55.0320	-	Adenine[b]	SM/CT
3	$C_5H_7NO_3$	1.23	130.0497 (-1.0)	-	84.0456$[M+H-HCOOH]^+$, 56.0527$[M+H-HCOOH-CO]^+$	-	L-Pyroglutamic acid	SM/CT
4	$C_9H_{12}N_2O_6$	1.33	245.0769 (0.5)	243.0622 (-0.2)	115.0403, 113.0355$[M+H-Ribose]^+$, 96.0084$[M+H-Ribose-NH_3]^+$, 85.0293, 70.0314$[M+H-Ribose-NH_3-C_2H_2]^+$, 57.0374	200.0584$[M-H-NH_3-C_2H_2]^-$, 152.0366, 140.0363,110.0264	Uridine[b]	SM/CT
5	$C_4H_4N_2O_2$	1.34	113.0349 (2.7)	111.0206 (5.1)	96.0102$[M+H-NH_3]^+$, 95.0234, 70.0318$[M+H-NH_3-C_2H_2]^+$, 68.0152$[M+H-NH_3-CO]^+$, 53.0057$[M+H-2NH_3-C_2H_2]^+$		Uracil[b]	SM/CT
6	$C_4H_6O_4$	1.38		117.0212 (6.2)		99.0109$[M-H-H_2O]^-$, 73.0317$[M-H-CO_2]^-$, 55.0219$[M-H-CO_2-H_2O]^-$	Succinic acid[b]	SM/CT
7	$C_6H_{13}NO_2$	1.38	132.1017 (-1.8)		86.0975$[M+H-HCOOH]^+$, 69.0718$[M+H-HCOOH-NH_3]^+$, 57.0605, 56.0525		Isoleucine[b]	SM
8	$C_6H_{13}NO_2$	1.48	132.1018 (-0.9)		86.0977$[M+H-HCOOH]^+$, 69.0721$[M+H-HCOOH-NH_3]^+$, 57.0606, 56.0526		Leucine	SM/CT
9	$C_{10}H_{13}N_5O_4$	1.68	268.1043 (1.2)	266.0891 (-1.3)	136.0624$[M+H-Ribose]^+$, 119.0358$[M+H-Ribose-NH_3]^+$	134.0478$[M-H-Ribose]^-$,107.0358	Adenosine[b]	SM/CT
10	$C_{10}H_{13}N_5O_5$	1.88	284.0985 (-1.6)	282.0836 (-2.7)	135.0296$[M+H-Ribose-NH_3]^+$, 110.0351	150.0418$[M-H-Ribose]^-$, 133.0155$[M-H-Ribose-NH_3]^-$, 108.0201	Guanosine	SM/CT
11	$C_7H_6O_5$	1.98		169.0148 (3.2)		125.0252$[M-H-CO_2]^-$, 124.0150, 79.0206$[M-H-CO_2-C_2H_4-H_2O]^-$, 53.0422	Gallic acid	NF
12	$C_9H_{11}NO_2$	2.55	166.0865 (1.5)	164.0726 (5.3)	120.0815$[M+H-H_2CO_2]^+$, 103.0555$[M+H-NH_3-H_2CO_2]^+$, 91.0557,77.0408	147.0450$[M-H-NH_3]^-$, 103.0560$[M-H-NH_3-CO_2]^-$,72.0099	Phenylalanine[b]	SM/CT
13	$C_6H_6O_3$	2.84	127.0390 (1.1)		109.0296$[M+H-H_2O]^+$, 81.0353$[M+H-CO]^+$, 53.0428$[M+H-2CO]^+$		5-Hydroxymethyl furfural	SM

NO	Formula	T_R (min)	[M+H]+ (error)	$[M-H]^-$ (error)	Fragment ions in positive (+) ion mode[a]	Fragment ions in negative (−) ion mode	Identification	Attribution
14	$C_{13}H_{16}O_8$	2.92	301.0903 (-4.8)	299.0775 (0.8)	283.0735$[M+H-H_2O]^+$, 139.0382$[M+H-Glc]^+$, 121.0288$[M+H-Glc-H_2O]^+$, 95.0495, 77.0392	137.0246$[M-H-Glc]^-$, 93.0352$[M-H-Glc-CO_2]^-$	p-Hydroxybenzonic acid-*O*-glucoside	CT
15	$C_8H_8O_4$	3.17	169.0495 (-0.1)	167.0357 (4.3)	151.0352$[M+H-H_2O]^+$, 133.0275, 123.0417$[M+H-CO]^+$, 77.0405	121.0274$[M-H-H_2O-CO]^-$, 93.0337, 65.0398	2-Hydroxy-3',4'-dihydroxyacetophenone	SM
16	$C_9H_{10}O_5$	3.25		197.0465 (2.3)		179.0352$[M-H-H_2O]^-$, 151.0403$[M-H-CO_2]^-$, 135.0455$[M-H-H_2O-CO_2]^-$, 72.9947	Danshensu[b]	SM
17	$C_7H_6O_4$	3.55	155.0339 (0.1)	153.0201 (4.8)	137.0227$[M+H-H_2O]^+$,111.0443$[M+H-CO_2]^+$, 93.0339$[M+H-H_2O-CO_2]^+$, 65.0405	109.0310$[M-H-CO_2]^-$, 108.0222, 91.0188$[M-H-H_2O-CO_2]^-$, 65.0053	Protocatechuic acid[b]	SM/CT
18	$C_8H_8O_4$	3.64		167.0353 (1.6)		123.0466$[M-H-CO_2]^-$, 93.0345, 65.0401	Vanillic acid[b]	NF
19	$C_{10}H_{12}O_5$	4.09		211.0605 (-3.3)		167.0347$[M-H-CO_2]^-$, 149.0598$[M-H-H_2O-CO_2]^-$, 123.0447	Danshensu methyl ester	NF
20	$C_{11}H_{12}N_2O_2$	4.38	205.0972 (0.2)	203.0826 (-0.2)	188.0700$[M+H-NH_3]^+$, 170.0593$[M+H-NH_3-H_2O]^+$, 159.0911$[M+H-HCOOH]^+$, 146.0599, 118.0650, 91.0550	159.0904$[M-H-CO_2]^-$, 142.0641$[M-H-CO_2-NH3]^-$, 130.0652, 116.0499$[M-H-CO_2-NH_3-C_2H_2]^-$, 74.0243	Tryptophane[b]	SM/CT
21	$C_9H_{10}O_4$	4.58		181.0512 (3.3)		163.0403$[M-H-H_2O]^-$, 135.0453$[M-H-H_2O-CO]^-$, 119.0500, 72.9939	3,4-Dihydroxybenzenepropionic acid	SM/CT
22	$C_7H_6O_3$	4.71	139.0390 (0.8)	137.0260 (11.8)	111.0448$[M+H-CO]^+$, 93.0347$[M+H-CO-H_2O]^+$, 65.0415	109.0302$[M-H-CO]^-$, 108.0225, 92.0282, 81.0360	Protocatechualdehyde[b]	SM
23	$C_{16}H_{18}O_9$	4.72	355.1015 (-2.5)	353.0858 (-5.6)		191.0552[Quinic acid-H]$^-$, 179.0333, 135.0440, 85.0299	Neochlorogenic acid	CT
24	$C_{16}H_{20}O_{10}$	5.29		371.0969 (-3.8)		325.0910$[M-H-H_2O-CO]^-$, 163.0402, 119.0505	4-(2-Carboxyethenyl)-2-(3,4-dihydroxyphenyl)-2,3-dihydro-7-hydroxy-3-methylester, [2α, 3β, 4(E)-3-benzofurancarboxylic acid	CT

NO	Formula	T_R (min)	[M+H]+ (error)	$[M-H]^-$ (error)	Fragment ions in positive (+) ion mode[a]	Fragment ions in negative (−) ion mode	Identification	Attribution
25	$C_{18}H_{14}O_8$	5.98	359.07631 (0.4)	357.0618 (0.6)	341.0654$[M+H-H_2O]^+$, 297.0789$[M+H-H_2O-CO_2]^+$, 279.0684$[M+H-2H_2O-CO_2]^+$,249.0501,233.0567	313.0728$[M-H-CO_2]^-$, 269.0822$[M-H-2CO_2]^-$, 253.0517, 225.0571$[M-H-C_2H_2-H_2O-2CO_2]^-$,	Prolithospermic acid	NF
26	$C_{15}H_{18}O_9$	6.15		341.0860 (-5.2)		179.0347$[M-H-Hexoside]^-$, 135.0454$[M-H-Hexoside-CO_2]^-$	Caffic acid-*O*-hexoside	SM/CT
27	$C_{15}H_{18}O_8$	6.26		325.0931 (0.7)		163.0398$[M-H-Hexoside]^-$, 119.0508$[M-H-Hexoside-CO_2]^-$	Coumaric acid-*O*-hexoside	CT
28	$C_{27}H_{32}O_{16}$	6.47	613.1766 (0.4)	611.1596 (-3.5)	451.1210$[M+H-Glc]^+$, 433.1118$[M+H-Glc-H_2O]^+$, 331.1060, 289.0755$[M+H-2Glc]^+$, 277.0483, 211.0239, 181.0088, 147.0455	521.1310$[M-H-C_3H_6O_3]^-$, 491.1145$[M-H-C_4H_8O_4]^-$, 359.0748$[M-H-C_4H_8O_4-C_5H_{10}O_5]^-$, 119.0508	Hydroxysafflor yellow A[b]	CT
29	$C_{10}H_{10}O_4$	6.50	195.0646 (-3.0)	193.0504 (-1.0)	177.0534$[M+H-H_2O]^+$, 149.0289$[M+H-H_2O-CO]^+$, 135.0375, 117.0310, 89.0380, 77.0399	149.0616$[M-H-CO_2]^-$, 121.0663$[M-H-CO_2-C_2H_4]^-$	Ferulic acid	SM
30	$C_{16}H_{18}O_9$	6.58	355.1019 (1.2)	353.0880 (0.4)	163.0387$[M+H-Quinic\ acid]^+$, 145.0283$[M+H-Quinic\ acid-H_2O]^+$, 117.0331$[M+H-Quinic\ acid-H_2O-CO]^+$	191.0553$[Quinic\ acid-H]^-$, 179.0344, 173.0449, 135.0444, 111.0455, 93.0341	Chlorogenic acid	CT
31	$C_{33}H_{40}O_{22}$	6.63	789.2086 (0.2)	787.1914 (-3.1)	627.1561$[M+H-Glc]^+$, 465.1032$[M+H-2Glc]^+$, 303.0498,	625.1397$[M-H-Glc]^-$, 463.0883$[M-H-2Glc]^-$, 299.0244	6-Hydroxykaempferol-tri-*O*-glucoside	CT
32	$C_{18}H_{26}O_{11}$	6.65		417.1399 (-0.9)		209.0795$[M-H-Glc-H_2O-CO]^-$, 194.0578	Syringin	SM/CT
33	$C_9H_8O_4$	6.66	181.0497 (0.7)	179.0354 (2.6)	163.0383$[M+H-H_2O]^+$, 139.0397, 135.0441$[M+H-H_2O-CO]^+$, 111.0451, 93.0348, 65.0413	135.0448$[M-H-CO_2]^-$, 134.0369	Caffeic acid[b]	SM
34	$C_{17}H_{26}O_{11}$	6.83		405.1392 (-2.4)		359.1345$[M-H-H_2O-CO]^-$, 197.0815$[M-H-H_2O-CO-Glc]^-$, 153.0898, 89.0240	Ixoroside	CT
35	$C_{27}H_{32}O_{16}$	7.07	613.1766 (0.4)	611.1596 (-3.5)		593.1450$[M-H-H_2O]^-$, 521.1219$[M-H-C_3H_6O_3]^-$, 491.1172$[M-H-C_4H_8O_4]^-$, 359.0748$[M-H-C_4H_8O_4-C_5H_{10}O_5]^-$, 328.0566	Hydroxysafflor yellow A isomer	CT
36	$C_{27}H_{30}O_{17}$	7.21	627.1560 (0.7)	625.1386 (-3.9)	465.1015$[M+H-Glc]^+$,303.0490$[M+H-2Glc]^+$	463.0802$[M-H-Glc]^-$, 287.0500	Quercetin-di-*O*-glucoside	CT

NO	Formula	T_R (min)	[M+H]+ (error)	$[M-H]^-$ (error)	Fragment ions in positive (+) ion mode[a]	Fragment ions in negative (−) ion mode	Identification	Attribution
37	$C_{27}H_{30}O_{17}$	7.45	627.1560 (0.7)	625.1389 (-3.3)	465.1008$[M+H-Glc]^+$, 303.0488$[M+H-2Glc]^+$	463.0881$[M-H-Glc]^-$, 301.0343$[M-H-2Glc]^-$, 271.0254	6-Hydroxykaempferol-di-*O*-glucoside isomer	CT
38	$C_{16}H_{22}O_9$	7.54		357.1183 (-2.3)		195.0659$[M-H-Glc]^-$, 136.0518, 119.0505	Sweroside	CT
39	$C_{27}H_{32}O_{16}$	7.60		611.1596 (-3.5)		521.1283$[M-H-C_3H_6O_3]^-$, 491.1197$[M-H-C_4H_8O_4]^-$, 328.0556	Hydroxysafflor yellow A isomer	CT
40	$C_{20}H_{32}O_{10}$	7.78		431.1921 (-0.5)		385.1873$[M-H-H_2O-CO]^-$, 223.1336$[M-H-H_2O-CO-Glc]^-$, 161.0451, 153.0913	Roseside	SM/CT
41	$C_{17}H_{26}O_{11}$	7.95		405.1401 (-0.2)		359.1331$[M-H-H_2O-CO]^-$	Morroniside	CT
42	$C_{18}H_{14}O_8$	8.06	359.0764 (0.7)	357.0599 (-4.7)	341.0658$[M+H-H_2O]^+$, 313.0725$[M+H-H_2O-CO]^+$, 295.0601$[M+H-2H_2O-CO]^+$, 267.0623$[M+H-2H_2O-2CO]^+$, 251.0724, 221.0562, 161.0602	313.0712$[M-H-CO_2]^-$, 269.0803$[M-H-2CO_2]^-$, 203.0327, 159.0443, 109.0284	Prolithospermic acid isomer	SM
43	$C_{27}H_{24}O_{13}$	8.25	557.1259 (-5.5)	555.1090 (-9.8)		511.1191$[M-H-CO_2]^-$, 493.1144$[M-H-CO_2-H_2O]^-$, 313.0761$[M-H-DS-CO_2]^-$, 295.0583$[M-H-DS-CO_2-H_2O]^-$, 197.0428$[DS-H]^-$,185.0234,109.0283	Salvianolic acid K isomer	NF
44	$C_9H_8O_3$	8.57	165.0547 (0.2)	163.0405 (2.7)	147.0446$[M+H-H_2O]^+$, 119.0494$[M+H-H_2O-CO]^+$, 91.0554$[M+H-H_2O-2CO]^+$, 65.0412	119.0498$[M-H-CO_2]^-$, 93.0349$[M-H-CO_2-C_2H_2]^-$, 65.0415	p-Coumaric acid[b]	CT
45	$C_{27}H_{24}O_{13}$	8.95	557.1292 (0.4)	555.1078 (-12)		511.1246$[M-H-CO_2]^-$, 493.11074, 295.0605$[M-H-DS-H_2O]^-$, 269.0821, 197.0439$[DS-H]^-$,185.0239	Salvianolic acid K isomer	SM
46	$C_{27}H_{30}O_{17}$	8.98	627.1559 (0.5)	625.1375 (-5.6)	465.1033$[M+H-Glc]^+$, 303.0498$[M+H-2Glc]^+$, 97.0290	463.0860$[M-H-Glc]^-$, 301.0355$[M-H-2Glc]^-$, 271.0264	6-Hydroxykaempferol-di-*O*-glucoside	CT
47	$C_{27}H_{30}O_{16}$	9.24	611.1610 (0.6)	609.1401 (-9.8)	449.1107$[M+H-Glc]^+$, 287.0561$[M+H-2Glc]^+$, 145.0685	447.0903$[M-H-Glc]^-$, 283.0233, 255.0293	Kaempferol-di-*O*-glucoside isomer	CT
48	$C_{27}H_{24}O_{13}$	9.56		555.1108 (-6.5)		511.1260$[M-H-CO_2]^-$, 493.1132$[M-H-CO_2-H_2O]^-$, 295.0616$[M-H-DS-CO_2-H_2O]^-$, 271.0623, 197.0435$[DS-H]^-$, 185.0241, 109.0300	Salvianolic acid K isomer	SM

NO	Formula	T_R (min)	[M+H]+ (error)	$[M-H]^-$ (error)	Fragment ions in positive (+) ion mode[a]	Fragment ions in negative (−) ion mode	Identification	Attribution
49	$C_{18}H_{12}O_7$	9.58	341.0659 (0.8)	339.0478 (-9.6)	323.0534$[M+H-H_2O]^+$, 295.0592$[M+H-H_2O-CO]^+$, 277.0494$[M+H-2H_2O-CO]^+$, 249.0552$[M+H-2H_2O-2CO]^+$, 221.0571$[M+H-2H_2O-3CO]^+$, 187.0348, 137.0222, 131.0493, 109.0283	295.0574$[M-H-CO_2]^-$, 253.0495, 225.0544	Salvianolic acid G isomer	CT
50	$C_{27}H_{24}O_{13}$	9.89		555.1108 (-6.5)		511.1262$[M-H-CO_2]^-$, 493.1119$[M-H-CO_2-H_2O]^-$, 313.0701$[M-H-DS-CO_2]^-$, 295.0610$[M-H-DS-CO_2-H_2O]^-$, 197.0438$[DS-H]^-$, 185.0240, 109.0289	Salvianolic acid K	SM
51	$C_{27}H_{30}O_{16}$	10.02	611.1612 (0.9)	609.1421 (-6.5)	449.0963$[M+H-Glc]^+$, 287.0552$[M+H-2Glc]^+$	429.0828$[M-H-Glc-H_2O]^-$, 285.0411$[M-H-2Glc]^-$, 255.0288,227.0331	Kaempferol-di-*O*-glucoside	CT
52	$C_{27}H_{22}O_{12}$	10.25	539.1185 (0.1)	537.1005 (-6.3)	521.1066$[M+H-H_2O]^+$, 323.0549$[M+H-H_2O-DS]^+$, 297.0759$[M+H-H_2O-DS-CO]^+$, 279.0648$[M+H-2H_2O-DS-CO]^+$, 233.0586$[M+H-3H_2O-DS-2CO]^+$, 139.0385	493.1151$[M-H-CO_2]^-$, 295.0612$[M-H-CO_2-DS]^-$, 185.0245, 109.0301	Salvianolic acid H	SM
53	$C_{27}H_{22}O_{12}$	10.60	539.1185 (0.1)	537.1005 (-6.3)	521.1073$[M+H-H_2O]^+$, 323.0551$[M+H-H_2O-DS]^+$, 297.0760$[M+H-H_2O-DS-CO]^+$, 279.0650$[M+H-2H_2O-DS-CO]^+$, 261.0540$[M+H-3H_2O-DS-CO]^+$, 233.0597$[M+H-3H_2O-DS-2CO]^+$, 139.0387	439.1145$[M-H-CO_2]^-$, 295.0608$[M-H-CO_2-DS]^-$, 185.0243, 109.0298	Salvianolic acid I	NF
54	$C_{24}H_{26}O_{13}$	10.82	523.1426 (-3.9)	521.1301 (0.1)	361.0929$[M+H-Glc]^+$, 163.0383$[M+H-Glc-DS]^+$, 145.0273$[M+H-Glc-DS-H_2O]^+$, 139.0394	359.0779$[M-H-Glc]^-$, 323.0777$[M-H-Glc-2H_2O]^-$, 179.0349$[M-H-Glc-CA]^-$, 161.0243$[M-H-Glc-DS]^-$, 135.0448	Salviaflaside	SM
55	$C_{21}H_{22}O_{11}$	10.85	451.1237 (0.4)	449.1020 (-5.4)	289.0712$[M+H-Glc]^+$, 169.0121$[M+H-Glc-C_4H_8O_4]^+$, 147.0405, 85.0300	287.0550$[M-H-Glc]^-$, 181.0146, 165.9903,153.0181	Isocarthamin	CT
56	$C_{20}H_{18}O_{10}$	11.30	419.0975 (0.6)	417.0809 (-4.3)	401.0780$[M+H-H_2O]^+$, 221.0454$[M+H-DS]^+$, 181.0475, 177.0559$[M+H-DS-CO_2]^+$, 147.0440, 131.0493	373.0924$[M-H-CO_2]^-$, 197.0465, 193.0521$[M-H-CO_2-CA]^-$, 179.0364, 175.0415$[M-H-CO_2-DS]^-$, 157.0314, 135.0476, 129.0372, 123.0477, 72.9971	Salvianolic acid D[b]	SM
57	$C_{27}H_{30}O_{15}$	11.39	595.1663 (0.9)	593.1433 (-3.3)	499.1064, 433.1113$[M+H-Glc]^+$, 287.0557$[M+H-Glc-Rha]^+$, 255.0837, 129.0554	327.0538, 285.0410$[M-H-Glc-Rha]^-$, 284.0317, 255.0282, 227.0363	Kaempferol-*O*-rutinoside	CT

NO	Formula	T_R (min)	[M+H]+ (error)	[M-H]$^-$ (error)	Fragment ions in positive (+) ion mode[a]	Fragment ions in negative (−) ion mode	Identification	Attribution
58	$C_{18}H_{12}O_7$	11.51	341.0658 (0.7)	339.0496 (-4.1)	323.0580[M+H-H_2O]$^+$, 297.0858[M+H-CO_2]$^+$, 281.0435, 203.0348, 187.0372	321.0396[M-H-H_2O]$^-$, 295.0608[M-H-CO_2]$^-$, 293.0465[M-H-H_2O-CO]$^-$, 279.0306, 277.0518, 251.0335, 223.0397, 195.0480	Salvianolic acid G	SM
59	$C_{28}H_{24}O_{12}$	11.78	553.1337 (-0.6)	551.1152 (-7.7)	293.0722, 263.1032, 181.0545	507.1254[M-H-CO_2]$^-$, 327.0882[M-H-CO_2-CA]$^-$, 309.0760, 294.0530, 277.0518, 197.0453[DS-H]$^-$, 185.0242, 179.0349, 135.0466	Monomethyl lithospermate	NF
60	$C_{27}H_{29}NO_{13}$	11.97	576.1713 (0.2)	574.1481 (-4.9)	558.1550[M+H-H_2O]$^+$, 414.1146[M+H-Glc]$^+$, 354.0842, 294.0587[M+H-Glc-$C_4H_8O_4$]$^+$, 248.0565, 246.0338, 244.0248, 234.0412, 178.0519, 147.0438	466.1072, 454.0925[M-H-$C_4H_8O_4$]$^-$, 364.0796[M-H-$C_3H_6O_3$-$C_4H_8O_4$]$^-$, 244.0229, 216.0309	Cartormin	CT
61	$C_{18}H_{16}O_8$	12.15		359.0761 (-3.1)		197.0463[DS-H]$^-$, 179.0356[M-H-CA]$^-$, 161.0251[M-H-CA-H_2O]$^-$, 135.0465, 123.0467, 72.9962	Rosmarinic acid[b]	SM
62	$C_{27}H_{22}O_{12}$	12.49	539.1184 (0.4)	537.1046 (1.5)	521.0935[M+H-H_2O]$^+$, 341.0665[M+H-DS]$^+$, 323.0560[M+H-H_2O-DS]$^+$, 295.0593[M+H-H_2O-DS-CO]$^+$, 279.0586, 249.0572, 179.0346, 135.0390	493.1098[M-H-CO_2]$^-$, 313.0697[M-H-CO_2-CA]$^-$, 295.0602[M-H-CO_2-DS]$^-$, 185.0250, 109.0310	Lithospermic acid[b]	SM
63	$C_{18}H_{16}O_5$	12.81	313.1078 (2.3)		295.0948[M+H-H_2O]$^+$, 277.0862[M+H-2H_2O]$^+$, 267.0650[M+H-H_2O-CO]$^+$, 225.0525, 197.0592, 169.0623, 153.0680		Tanshindiol A	SM
64	$C_{37}H_{32}O_{16}$	13.21		731.1618 (0.0)		551.1216[M-H-CA]$^-$, 533.1105[M-H-DS]$^-$, 353.0654[M-H-DS-CA]$^-$, 335.0559[M-H-2DS]$^-$, 309.0719	9''-Methyl lithospermate B	NF
65	$C_{36}H_{30}O_{16}$	13.46	719.1608 (0.4)	717.1482 (2.9)	521.1042[M+H-DS]$^+$, 341.0661[M+H-DS-CA]$^+$, 323.0540[M+H-2DS]$^+$, 295.0595[M+H-2DS-CO]$^+$, 181.0490[CA+H]$^+$	519.0887[M-H-DS]$^-$, 339.0498[M-H-DS-CA]$^-$, 321.0299[M-H-2DS]$^-$	Salvianolic acid E	SM/CT
66	$C_{36}H_{30}O_{16}$	13.89	719.1581 (-3.6)	717.1485 (3.3)		519.0932[M-H-DS]$^-$, 339.0510[M-H-DS-CA]$^-$, 321.0409[M-H-2DS]$^-$, 295.0596	Salvianolic acid B[b]	SM
67	$C_{29}H_{26}O_{12}$	13.95		565.1352 (0.1)		519.0909[M-H-CH_3CH_2OH]$^-$, 367.0823[M-H-DS]$^-$, 321.0417[M-H-DS-CH_3CH_2OH]$^-$, 293.0431[M-H-DS-CH_3CH_2OH-CO]$^-$, 245.0453, 109.0286	Ethyl lithospermate	NF

NO	Formula	T_R (min)	[M+H]+ (error)	$[M-H]^-$ (error)	Fragment ions in positive (+) ion mode[a]	Fragment ions in negative (−) ion mode	Identification	Attribution
68	$C_{36}H_{30}O_{16}$	14.11	719.1587 (-2.7)	717.1490 (4.1)	521.1069$[M+H-DS]^+$, 323.0549$[M+H-2DS]^+$, 295.0607$[M+H-2DS-CO]^+$, 181.0491$[CA+H]^+$	519.0940$[M-H-DS]^-$, 339.0520$[M-H-DS-CA]^-$, 321.0410$[M-H-2DS]^-$, 295.0619, 185.0246	Salvianolic acid L	SM
69	$C_{19}H_{18}O_8$	14.35		373.0923 (-1.6)		179.0336$[CA-H]^-$, 135.0446	Romarinic acid methyl ester	SM
70	$C_{26}H_{22}O_{10}$	14.45		493.1145 (0.9)		313.0704$[M-H-CA]^-$, 295.0594$[M-H-DS]^-$, 185.0250$[M-H-DS-C_6H_6O_2]^-$, 159.0455$[M-H-DS-C_6H_6O_2-C_2H_2]^-$, 135.0457, 109.0306	Salvianolic acid A[b]	SM
71	$C_{18}H_{16}O_5$	14.66	313.1071 (2.3)		295.0944$[M+H-H_2O]^+$, 277.0874$[M+H-2H_2O]^+$, 267.0979$[M+H-H_2O-CO]^+$, 249.0904$[M+H-2H_2O-CO]^+$, 169.0647, 151.1099, 95.0464		Tanshindiol B	SM
72	$C_{37}H_{32}O_{16}$	14.79		731.1628 (1.4)		533.1121$[M-H-DS]^-$, 353.0656$[M-H-DS-CA]^-$, 335.0561$[M-H-2DS]^-$, 309.0742	9''-Methyl lithospermate B/isomer	SM
73	$C_{26}H_{22}O_{10}$	14.96	495.1279 (-1.4)	493.1144 (0.7)	269.0822$[M+H-DS-CO]^+$, 251.0695$[M+H-DS-CO-H_2O]^+$, 223.0742$[M+H-DS-2CO-H_2O]^+$, 205.0655, 181.0448, 143.0461, 139.0379, 135.0457, 111.0401	313.0704$[M-H-CA]^-$, 295.0613$[M-H-DS]^-$, 185.0240$[M-H-DS-C_6H_6O_2]^-$, 159.0448$[M-H-DS-C_6H_6O_2-C_2H_2]^-$, 109.0297	Salvianolic acid A isomer	NF
74	$C_{26}H_{20}O_{10}$	15.46	493.1129 (0.6)	491.0989 (1.0)	313.0688$[M+H-CA]^+$, 295.0604$[M+H-DS]^+$, 267.0696, 135.0178	311.0560$[M-H-CA]^-$, 293.0454$[M-H-DS]^-$, 265.0505$[M-H-DS-CO]^-$, 249.0562$[M-H-DS-CO_2]^-$, 197.0444$[DS-H]^-$, 135.0457	Salvianolic acid C isomer	SM
75	$C_{29}H_{26}O_{12}$	15.80		565.1352 (-0.7)		519.0821$[M-H-CH_3CH_2OH]^-$, 367.0766$[M-H-DS]^-$, 321.0389$[M-H-DS-CH_3CH_2OH]^-$, 293.0477$[M-H-DS-CH_3CH_2OH-CO]^-$, 245.0424, 197.0503, 135.0460, 109.0313	Ethyl lithospermate isomer	NF
76	$C_{26}H_{20}O_{10}$	16.75		491.0986 (0.5)		311.0529$[M-H-CA]^-$, 293.0433$[M-H-DS]^-$, 265.0467$[M-H-DS-CO]^-$, 249.0433$[M-H-DS-CO_2]^-$, 247.0391$[M-H-DS-CO-H_2O]^-$, 135.0447	Salvianolic acid C	SM

NO	Formula	T_R (min)	[M+H]+ (error)	$[M-H]^-$ (error)	Fragment ions in positive (+) ion mode[a]	Fragment ions in negative (−) ion mode	Identification	Attribution
77	$C_{18}H_{18}O_4$	17.49	299.1282 (1.4)		281.1169$[M+H-H_2O]^+$, 263.1067$[M+H-2H_2O]^+$, 251.1059, 235.1104$[M+H-2H_2O-CO]^+$, 192.0918, 165.0687		Phenanthro[1,2-b] furan-10,11-dione , 1,2,6,7,8,9-hexah ydro-6-hydroxy-1 ,6-dimethyl-	SM
78	$C_{19}H_{20}O_4$	17.91	313.1434 (0.2)		295.1247$[M+H-H_2O]^+$, 269.1547, 267.1358$[M+H-H_2O-CO]^+$,253.0864, 249.1241, 225.2208$[M+H-H_2O-CO-C_3H_6]^+$, 199.0749, 197.1349$[M+H-H_2O-2CO-C_3H_6]^+$, 171.0818, 169.1022, 155.1316, 141.0675, 128.0635, 115.1976		17-Hydroxycrypt otanshinone	SM
79	$C_{19}H_{20}O_3$	17.99	297.1485 (1.0)		253.1579, 251.14238$[M+H-H_2O-CO]^+$, 237.0906, 211.1104, 181.1008, 165.0694, 155.0847, 141.0701, 128.0617		Unknown tanshinone	SM
80	$C_{19}H_{20}O_4$	18.42	313.1437 (0.8)		295.1354$[M+H-H_2O]^+$, 277.1546, 267.1346$[M+H-H_2O-CO]^+$, 249.1302, 225.2198$[M+H-H_2O-CO-C_3H_6]^+$, 197.1333$[M+H-H_2O-2CO-C_3H_6]^+$		Hydroxycryptotan shinone isomer	SM
81	$C_{19}H_{20}O_4$	20.27	313.1435 (1.3)		295.1317$[M+H-H_2O]^+$, 277.1532$[M+H-2H_2O]^+$, 249.1198$[M+H-2H_2O-CO]^+$, 225.1040		3-Hydroxycryptot anshinone	SM
82	$C_{19}H_{20}O_3$	23.09	297.1487 (0.5)		269.1538$[M+H-CO]^+$, 253.1577, 251.1426$[M+H-H_2O-CO]^+$, 237.0904, 223.1109$[M+H-H_2O-2CO]^+$, 211.1130, 181.1000, 165.0690, 141.0692, 128.0618		Unknown tanshinone	SM

[a] DS: Danshensu; CA: Caffic acid; Glc: Glucoside; SM: Salvia Miltiorrhiza; CT: Carthamus tinctorious; NF: newly formed compounds.

[b] Confirmation in comparison with authentic standards.

Supplementary Table S2. Weights and ratio of SM and CT herbs in the nine DHI prescription modified samples under uniform design. SM, Salvia Miltiorrhiza; CT, Carthamus tinctorious. Sample S3 was the original formula.

No.	SM (g)	CT (g)	SM:CT
S1	400	0	100：0
S2	350	50	7：1
S3	300	100	3：1
S4	250	150	5：3
S5	200	200	1：1
S6	150	250	3：5
S7	100	300	1：3
S8	50	350	1：7
S9	0	400	0：100

Supplementary Table S3. Dimensionless data of peak areas of 13 identified components in nine DHI modified samples. S1-S9 represented nine DHI samples. P1-P13 represented 13 identified components in DHI and were regarded as the independent ingredient variables. P1-P13 were processed to be dimensionless by the equalization method for the calculation.

No.	P1	P2	P3	P4	P5	P6	P7	P8	P9	P10	P11	P12	P13
S1	0	1.57	1.93	0	0	2.08	2.07	2.12	1.52	1.97	1.34	1.41	0.58
S2	0	1.73	1.77	0	0	2.31	2.22	2.34	0.95	0.8	1.4	1.22	0.38
S3	0.37	1.98	2.44	1.1	1.5	2.01	2.35	2.28	2.3	1.94	2.82	2.46	3.88
S4	1.54	1.41	1.4	1.6	1.48	0.91	0.85	0.91	2.26	2.26	1.8	2.39	3.08
S5	2.72	1.21	0.47	0.39	0.18	0.55	0.45	0.47	0.45	0.45	0.6	0.41	0.21
S6	1.11	0.46	0.27	1.66	0.56	0.61	0.33	0.38	0.4	0.73	0.32	0.47	0.28
S7	0.58	0.47	0.47	1.03	1.59	0.35	0.42	0.32	0.92	0.66	0.56	0.47	0.46
S8	1.06	0.17	0.25	1.05	1.65	0.18	0.29	0.17	0.2	0.21	0.16	0.19	0.14
S9	1.62	0	0	2.17	2.05	0	0	0	0	0	0	0	0

Supplementary Table S4. Dimensionless data of pharmacological indexes of nine DHI modified samples. S1-S9 represented the nine DHI modified samples. Effect values of switched parameters were processed to be dimensionless by an equalization method for the calculation.

No.	WBV5	WBV50	WBV200	PV	MPAR	PT	APTT	EAI	ERI	RCEI
S1	1.0408	1.0208	1.0135	0.9595	1.0432	1.0040	0.9361	1.0240	1.0491	1.0042
S2	1.0653	1.0453	1.0317	0.9912	1.0276	1.0024	0.9507	1.0452	1.0611	1.0925
S3	1.0478	1.0690	1.0481	0.9912	0.9658	1.0114	0.9945	1.0240	1.0491	1.0313
S4	1.0065	1.0140	1.0271	0.9995	0.8642	0.9998	0.9714	1.0310	1.0759	1.0228
S5	1.0455	1.0242	1.0271	1.0079	0.8286	0.9934	1.0612	1.0137	0.9994	0.9596
S6	0.9249	0.9613	0.9708	0.9995	1.1950	0.9838	1.0298	0.9266	0.9444	0.9432
S7	1.0241	1.0123	0.9917	0.9831	0.8920	1.0072	0.9523	1.0171	0.9701	0.9962
S8	0.9603	0.9420	0.9508	1.0251	0.9422	0.9934	1.0390	0.9744	0.9219	0.9962
S9	0.8846	0.9111	0.9392	1.0429	0.9333	0.9955	1.0651	0.944	0.9146	0.9541

Supplementary Table S5. The rotating component matrix results between ten pharmacological indexes and five factors. F1-F5 represented Factor 1-5 respectively. Variance contribution means how much the corresponding factor reflects the original data. The larger the absolute value, the higher the relevance between indexes and factors.

Indexes	F1	F2	F3	F4	F5
WBV200	0.950	0.264	0.048	0.075	0.095
WBV50	0.904	0.345	0.093	0.168	-0.093
WBV5	0.842	0.369	0.016	0.301	-0.106
ERI	0.775	0.390	0.233	0.196	0.362
EAI	0.717	0.541	-0.124	0.363	0.079
RCEI	0.373	0.867	0.012	0.110	0.225
PT	0.413	0.840	0.010	0.200	-0.217
MPAR	-0.024	-0.026	0.989	0.092	0.030
PV	-0.560	-0.156	-0.578	-0.562	0.092
APTT	-0.307	-0.553	-0.392	-0.651	-0.100

Supplementary Table S6. Linear equation, R^2, and range of eight components for quantitative determination.

Compound	Regression equation	R^2	Range (μg/mL)
Danshensu	y=0.0147x+0.0648	1.0000	26.12-783.6
Hydroxysafflor yellow A	y=0.0404x+0.0430	0.9995	4.35-130.4
p-Coumaric acid	y=0.314x+0.0082	1.0000	1.31-39.40
Salvianolic acid D	y=0.0591x−0.0150	1.0000	14.4-432.2
Rosmarinic acid	y=0.0715x+0.0055	1.0000	7.57-227.1
Lithospermic acid	y=0.0136x+0.0360	1.0000	3.30-98.98
Salvianolic acid B	y=0.0259x-0.0818	0.9999	24.05-721.5
Salvianolic acid A	y=0.0325x+0.0850	0.9999	24.46-366.9

Supplementary Table S7. Contents of 8 components in DHI and CBC.

No.	Compound name	DHI Peak area (mAU*min)	DHI content (mg/10mL)	CBC peak area (mAU*min)	CBC content (mg/10mL)
2	Danshensu	8.4130	5.67	8.2528	5.57
4	Hydroxysafflor yellow A	0.5282	0.12	0.4883	0.11
5	p-Coumaric acid	2.5271	0.08	2.6520	0.08
8	Salvianolic acid D	2.8816	0.49	2.7463	0.47
10	Rosmarinic acid	5.5137	0.77	5.3918	0.75
11	Lithospermic acid	0.7157	0.50	0.7094	0.50
12	Salvianolic acid B	5.0020	1.96	5.0701	1.99
13	Salvianolic acid A	2.9743	0.89	2.9724	0.89

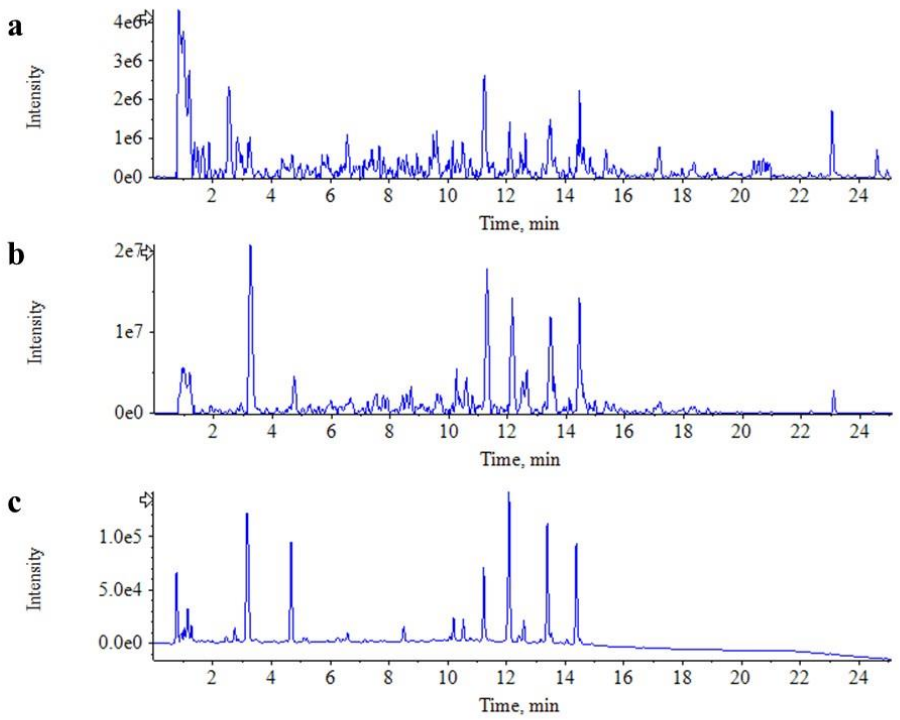

Supplementary Figure S1. Representative total ion chromatogram (TIC) of DHI in positive ion mode (a), negative ion mode (b) and PDA (c) by UFLC-PDA-Triple TOF-MS/MS.

Supplementary Vedio S1. The blood flow of cerebral microarterioles thirty minutes after irradiation in Control group. In control group, the blood flow velocity of the blocked vessel (the lower vessel in the video) reduced significantly and stagnated at 30 min.

Supplementary Vedio S2. The blood flow of cerebral microarterioles thirty minutes after irradiation in DHI group. In DHI group, the blood flow maintain at a relevant faster velocity. The clots were hardly to develop even 30 min after irradiation.
